# Genome-Wide Association Study for Milk Protein Composition Traits in a Chinese Holstein Population Using a Single-Step Approach

**DOI:** 10.3389/fgene.2019.00072

**Published:** 2019-02-19

**Authors:** Chenghao Zhou, Cong Li, Wentao Cai, Shuli Liu, Hongwei Yin, Shaolei Shi, Qin Zhang, Shengli Zhang

**Affiliations:** Key Laboratory of Animal Genetics, Breeding and Reproduction, Ministry of Agriculture & National Engineering Laboratory for Animal Breeding, College of Animal Science and Technology, China Agricultural University, Beijing, China

**Keywords:** genome-wide association, milk protein, casein, α-lactalbumin, β-lactoglobulin, ssGBLUP

## Abstract

Genome-wide association studies (GWASs) have been widely used to determine the genetic architecture of quantitative traits in dairy cattle. In this study, with the aim of identifying candidate genes that affect milk protein composition traits, we conducted a GWAS for nine such traits (α_s1_-casein, α_s2_-casein, β-casein, κ-casein, α-lactalbumin, β-lactoglobulin, casein index, protein percentage, and protein yield) in 614 Chinese Holstein cows using a single-step strategy. We used the Illumina BovineSNP50 Bead chip and imputed genotypes from high-density single-nucleotide polymorphisms (SNPs) ranging from 50 to 777 K, and subsequent to genotype imputation and quality control, we screened a total of 586,304 informative high-quality SNPs. Phenotypic observations for six major milk proteins (α_s1_-casein, α_s2_-casein, β-casein, κ-casein, α-lactalbumin, and β-lactoglobulin) were evaluated as weight proportions of the total protein fraction (wt/wt%) using a commercial enzyme-linked immunosorbent assay kit. Informative windows comprising five adjacent SNPs explaining no < 0.5% of the genomic variance per window were selected for gene annotation and gene network and pathway analyses. Gene network analysis performed using the STRING Genomics 10.0 database revealed a co-expression network comprising 46 interactions among 62 of the most plausible candidate genes. A total of 178 genomic windows and 194 SNPs on 24 bovine autosomes were significantly associated with milk protein composition or protein percentage. Regions affecting milk protein composition traits were mainly observed on chromosomes BTA 1, 6, 11, 13, 14, and 18. Of these, several windows were close to or within the *CSN1S1, CSN1S2, CSN2, CSN3, LAP3, DGAT1, RPL8*, and *HSF1* genes, which have well-known effects on milk protein composition traits of dairy cattle. Taken together with previously reported quantitative trait loci and the biological functions of the identified genes, we propose 19 novel candidate genes affecting milk protein composition traits: *ARL6, SST, EHHADH, PCDHB4, PCDHB6, PCDHB7, PCDHB16, SLC36A2, GALNT14, FPGS, LARP4B, IDI1, COG4, FUK, WDR62, CLIP3, SLC25A21, IL5RA*, and *ACADSB*. Our findings provide important insights into milk protein synthesis and indicate potential targets for improving milk quality.

## Introduction

Milk products are a fundamental component of many diets. Given the increasing development and variety of milk products, manufacturers, and scholars alike have placed a focus on understanding milk protein content and the importance of various milk proteins. The primary protein components of milk include α_s1_-casein (α_*s*1_-CN), α_s2_-casein (α_s2_-CN), β-casein (β-CN), κ-casein (κ-CN), α-lactalbumin (α-LA), and β-lactoglobulin (β-LG), each of which plays different roles in protein synthesis and metabolism in the human body. For example, casein intake affects vascular health (Fekete et al., 2013) and heart health (Miluchová et al., 2013), improves sleep quality (Brennan et al., 2013), and enhances immunity (Konstantinou et al., 2014).

Milk protein composition is a complex trait that is influenced by both genetic and non-genetic factors, including cattle breed, herd, and stage of lactation. Previous studies have shown that bovine milk protein composition is heritable, with heritability estimates ranging from 0.26 to 0.86 (Schopen et al., 2011) and 0.05 to 0.77 (Huang et al., 2012). In recent years, a number of genes and quantitative trait loci (QTL) for milk composition traits have been detected using candidate gene and QTL mapping methods. The effects of milk protein variants on α_s1_-CN, α_s2_-CN, β-CN, κ-CN, α-LA, and β-LG content have been examined in a number of studies (Heck et al., 2009; Sanchez et al., 2017; Viale et al., 2017). Variants of the β-CN and κ-CN genes located on bovine chromosome (BTA) 6 and variants of the β-LG gene located on BTA 11 have been associated with alterations in milk protein composition (Heck et al., 2009). A further β-LG protein variant has been associated with higher casein content (Lundén et al., 1997; Heck et al., 2009) and a higher cheese yield (Tsiaras et al., 2005). A previously reported genome-wide linkage study identified important QTLs for milk protein composition and content on BTA 1, 5, 6, 10, and 14 (Schopen et al., 2011).

Since the first application of genome-wide association studies (GWASs) to livestock research in 2008 (Daetwyler et al., 2008), a series of GWASs have been published on important economic traits. Such studies are of particular value with respect to livestock species, for which pedigrees are complex and nuclear families are the exception rather than the rule. Misztal et al. (2009) and Christensen and Lund (2010) proposed a single-step genomic best linear unbiased prediction (ssGBLUP) method that incorporates phenotypes, genotypes, and pedigree information. The use of this information in conjunction with genomic data allows more precise estimations and increased detection power through implementation of a scaled and properly augmented relationship matrix (Legarra et al., 2009; Misztal et al., 2009). Compared with multiple-step approaches, the ssGBLUP method yields more accurate and consistent solutions (Forni et al., 2011; Wang et al., 2012, 2014). In the present study, we applied the ssGBLUP method to identify genomic regions affecting bovine milk composition and protein content in the Chinese Holstein cow.

## Materials and Methods

### Animals and Phenotypes

The Chinese Holstein population used in this study included 614 cows from 19 farms of the Beijing Sanyuan Dairy Farm Center and the offspring of 19 sire families. For most individuals, we had access to both genotype data and traditional pedigree information. Genealogical information was available for all individuals and 598 individuals were genotyped. A total of 50 mL of milk was collected from each cow by the Dairy Herd Improvement System (DHI) laboratory of the Beijing Dairy Cattle Center. Samples were transported to the laboratory and stored at −20°C until use (Li et al., 2014). The concentrations of α_s1_-CN, α_s2_-CN, β-CN, κ-CN, α-LA, and β-LG in each sample were quantified using commercial ELISA kits in accordance with manufacturer instructions and expressed as the weight proportion of total protein (wt/wt%). Furthermore, protein percentage data were obtained from DHI reports and the casein index was calculated as [Σ casein/(Σ casein + Σ whey)] × 100 (Schopen et al., 2009).

### Genotyping, Imputation, and Quality Control

Genotyping was performed using one of two versions of the Illumina BovineSNP50 BeadChip (Illumina Inc., San Diego, CA, USA). Version 1 contains 54,001 SNPs and version 2 contains 54,609 SNPs. In order to improve the accuracy of the study results, we imputed genotypes from high-density (HD) single-nucleotide polymorphisms (SNPs) ranging from 50 to 777 K using BEAGLE version 3.3.1 (Browning and Browning, 2007). The data used in imputation included those for 85 Chinese Holstein bulls genotyped with both 54 and 777 K (HD) chips, 598 Chinese cows genotyped with a 54 K chip, and 510 Nordic Holstein bulls genotyped with an HD chip. This analysis enabled us to validate the imputation accuracy for the Chinese Holstein population in seven scenarios for cows and bulls using different reference populations (Ma et al., 2014). Following genotype imputation, the panel included a total of 644,400 SNPs. We excluded SNPs with a < 90% genotype call rate, minor allele frequency (MAF) < 0.05, and an absence of Hardy–Weinberg equilibrium (*P* < 10^−6^). Subsequent to quality control, a total of 586,304 SNPs were used for the association study. The position of each SNP was determined using the reference bovine genome sequence UMD_3.1.66 (http://www.ncbi.nlm.nih.gov/genome/guide/cow/).

### Genome-Wide Association Study

We conducted an association study in accordance with the single-step genomic-BLUP approach (Aguilar et al., 2010; Christensen and Lund, 2010; Misztal et al., 2013a). The Bayesian inference method was used to estimate variance components, and a Monte Carlo Markov Chain was completed for 100,000 rounds with Gibbs sampling, of which the first 9,000 rounds were discarded as burn-in. Within each Gibbs sample cycle, Metropolis–Hastings samples were run for 20 iterations. Trace plots were also inspected visually to ensure convergence had been reached. BLUPf90 family software was used to perform related analyses (Tiezzi et al., 2015; Parker Gaddis et al., 2018). The ssGBLUP model used was as follows:

$$\begin{array}{l} \text{y}=\text{X}\beta +\text{Wa}+\text{e}, \end{array}$$

where y is the observation vector; X represents the corresponding incidence matrix for fixed effects; β is a vector of fixed effects, including overall mean, farm, lactation, and parity; W represents a corresponding incidence matrix for random additive genetic effects; a is the vector of additive genetic effects; and e is a vector of residuals. The genetic variance and residual variance were calculated using the following formula:

$$\begin{array}{l} var\left[\begin{array}{c} a\\ e \end{array}\right]=\left[\begin{array}{cc} H\sigma_{a}^{2} & 0\\ 0 & I\sigma_{e}^{2} \end{array}\right], \end{array}$$

where $\sigma_{a}^{2}$ and $\sigma_{e}^{2}$ are the total additive genetic variance and residual variance, respectively. The model accounted for additive genetic relationships among different individuals and the pedigree as well as genomic information by integration into matrix H (Misztal et al., 2013b):

$$\begin{array}{l} H^{-1}=A^{-1}+\left[\begin{array}{cc} 0 & 0\\ 0 & G^{-1}-A_{22}^{-1} \end{array}\right] \end{array}$$

where A is a numerator (pedigree) relationship matrix applied for all animals, and A_22_ is a numerator (pedigree) relationship matrix applied for genotyped animals. G represents a genomic relationship matrix. Matrix G assumed the allele frequency of the current population and was adjusted for compatibility with A_22_ (Vanraden et al., 2009):

$$\begin{array}{l} G=ZDZ^{-1} \end{array}$$

where D represents a diagonal matrix in which elements contain the inverse of the expected maker variance (*D* = *I* for GBLUP) and Z represents a matrix containing genotypes under the correction of allele frequency (Prado et al., 2003). The animal effects of genotyped animals were a function of SNP effects:

$$\begin{array}{l} {\text{a}}_{\text{g}}=\text{Zu} \end{array}$$

where a_g_ represents animal effects decomposed in genotype, u denotes a vector of marker effects, and Z is a matrix related to the genotype of each locus. Thus, the variance of animal effects is expressed as

$$\begin{array}{l} \text{var}\left({\text{a}}_{\text{g}}\right)=\text{var}\left(\text{Zu}\right)={\text{ZDZ}}^{\prime }\sigma_{u}^{2}\;={\text{G}}^{*}\sigma_{\text{a}}^{2}, \end{array}$$

and the genetic additive variance can be captured by each SNP marker provided that the weighted relationship matrix (G^*^) is not weighted. Subsequently,

$$\begin{array}{l} {\text{G}}^{*}={\text{ZDZ}}^{\prime }\lambda , \end{array}$$

where λ is a normalization constant or variance ratio. Following Vanraden et al. (2009), we defined λ as follows:

$$\begin{array}{l} \lambda =\frac{\sigma_{u}^{2}}{\sigma_{a}^{2}}\;=\frac{1}{\sum_{i-1}^{M}2p_{i}\left(1-p_{i}\right)}, \end{array}$$

where $\sigma_{u}^{2}$ is the genetically additive variance captured by each SNP marker provided that G^*^ is not weighted, $\sigma_{a}^{2\;}$ is the overall genetic additive effect, M is the quantity of SNPs, and p_i_ is the allele frequency of the 2nd allele of the ith marker. SNP effects and the individual variance of each SNP were obtained using the following equation as described by Zhang et al. (2010):

$$\begin{array}{l} û=\lambda {\text{DZ}}^{\prime }G^{{\;}^{*}-1}{â}_{g}={\text{DZ}}^{\prime }{\left[ZDZ^{\prime }\right]}^{-1}{â}_{g},\\ {\hat{\sigma }}_{u,i}^{2}={\hat{\sigma }}_{u}^{2}2p_{i}\left(1-p_{i}\right). \end{array}$$

Wang et al. (2012) have previously described the “Scenario 1” process for iterative re-weighting. In the first round of the iterative process in the above formulae, we used D = I to predict SNP effects and the variance of each SNP by virtue of G^*^. In this study, the procedure was run for one iteration based on the realized accuracies of GEBV according to Wang et al. (2012). The weighted SNPs were used to construct the G matrices, update the GEBV, and, consequently, the estimated SNP effects. New marker effects were calculated in continuous iterations on the basis of the weighted G^*^ matrix proposed in the abovementioned formula.

The percentage of genetic variance explained by the i-th region was calculated as follows:

$$\begin{array}{l} \frac{Var\left(a_{i}\right)}{\sigma_{a}^{2}}\times 100\;=\frac{Var\left(\sum_{j=1}^{10}Z_{J}{û}_{j}\right)}{\sigma_{a}^{2}}\;\times 100 \end{array}$$

where a_i_ is the genetic value of the i-th region that consists of five continuous adjacent SNPs, $\sigma_{\text{a}}^{2}$ is the total genetic variance, Z_j_ is the vector of gene content of the j-th SNP for all individuals, and û_j_ is the marker effect of the i-th SNP within the i-th region (Zhang et al., 2010).

A significance test for SNP effects was performed using a two-sided *t*-test, and the *P*-value of each SNP was calculated as follows:

$$\begin{array}{l} P_{i}=P_{t}\left(\frac{{û}_{i}}{\sqrt{{\hat{\sigma }}_{i}^{2}/n}},n-1\right) \end{array}$$

where *P*_*t*_ is the distribution function of t distribution, û_*i*_ is the ith SNP effect, ${\hat{\sigma }}_{i}^{2}$ is the genetic variance of the i^th^ SNP, *n* is the number of animals with the i^th^ SNP. A Bonferroni correction was applied to control for false positive associations, and the genome significance level was defined as *P* < 0.01/N, where N is the number of SNP loci analyzed. Thus, in the present study, the significance threshold value of –log_10_(P) for all studied traits was 6.52 (586,304 SNP markers) (Wu et al., 2017).

### Gene Functional Annotation, Gene Network, and Pathway Analyses

The successive calculation of variance absorbed by 5-SNP moving windows was based on the whole genome. Windows explaining no < 0.5% of the genomic variance were selected for gene annotation, network, and pathway analyses (Fragomeni et al., 2014; Medeiros de Oliveira Silva et al., 2017). We used the Biomart platform of Ensemble (Flicek et al., 2013) to obtain gene annotations through the Biomart R package on the basis of the starting and ending coordinates of each window (http://www.bioconductor.org). A pathway-enrichment analysis, visualization, and integrated discovery (DAVID) analysis (Huang Da et al., 2009a,b) was performed using the Kyoto Encyclopedia of Genes and Genomes (KEGG) database (Kanehisa et al., 2014) for annotation. Manhattan plots of genome-wide association analyses were produced in R using the CMplot package. For candidate genes, we investigated functional protein–protein interactions (PPIs) and the enrichment of gene ontology (GO) using the STRING Genomics 10.0 database (Szklarczyk et al., 2015). This analysis evaluated two types of PPI: PPIs obtained from laboratory and curated databases and predicted PPIs based on gene neighborhood, fusion, gene co-occurrence, protein homology, co-expression, or text mining in the literature. A global PPI network was constructed and limited to interactions exhibiting high confidences with scores > 0.4.

## Results

**Figure 1:**
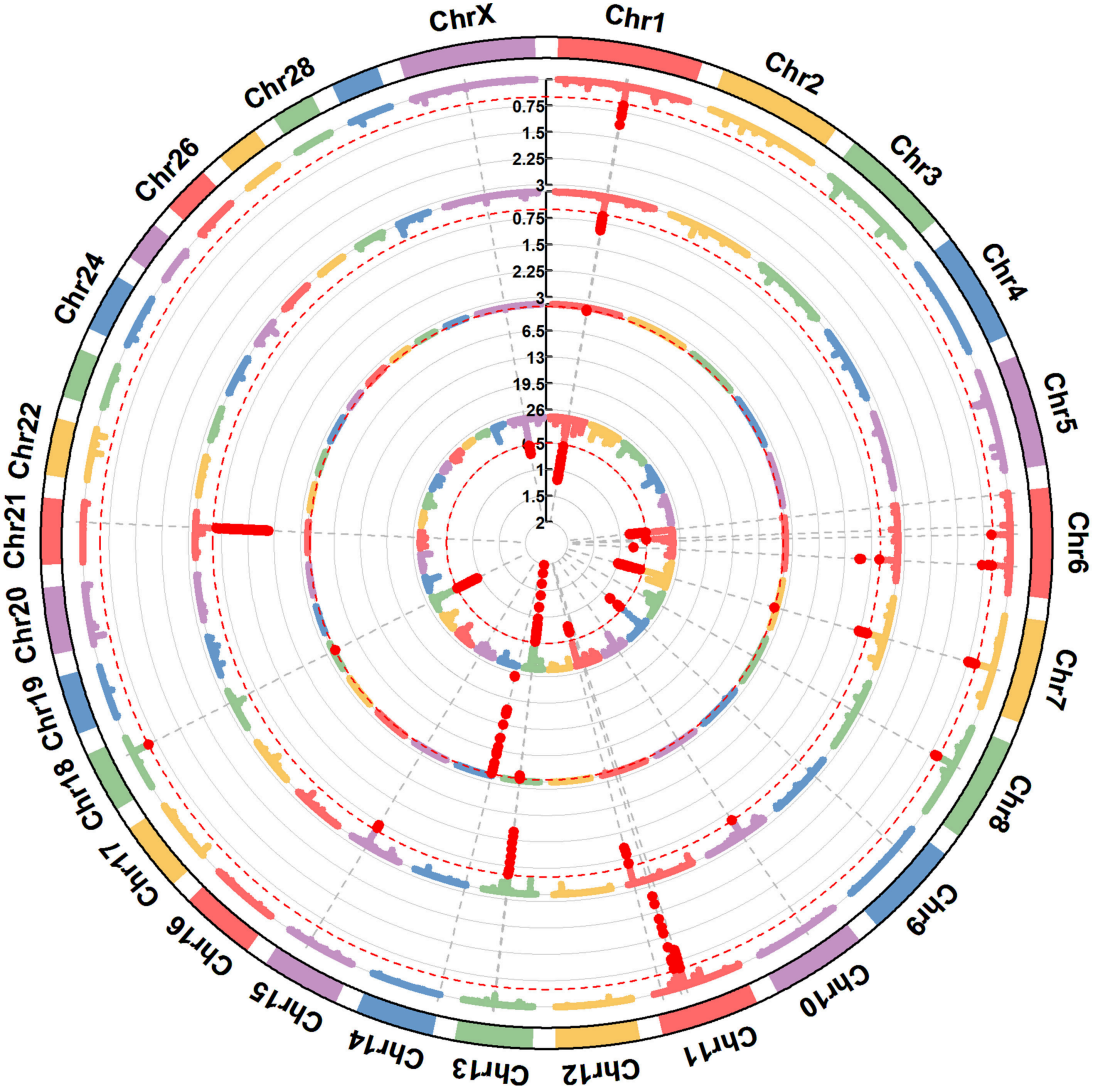
A circular-Manhattan plot for the proportion of genetic variance explained by the 5-SNP moving windows associated with the milk protein composition traits α_s1_-CN, α_s2_-CN, β-CN, and κ-CN. The horizontal line represents windows explaining no < 0.5% of the genomic variance. The four milk protein composition traits were plotted from inside to outside, respectively. A rectangular-Manhattan version of the plot is shown in the Supplementary Figures.

**Figure 2:**
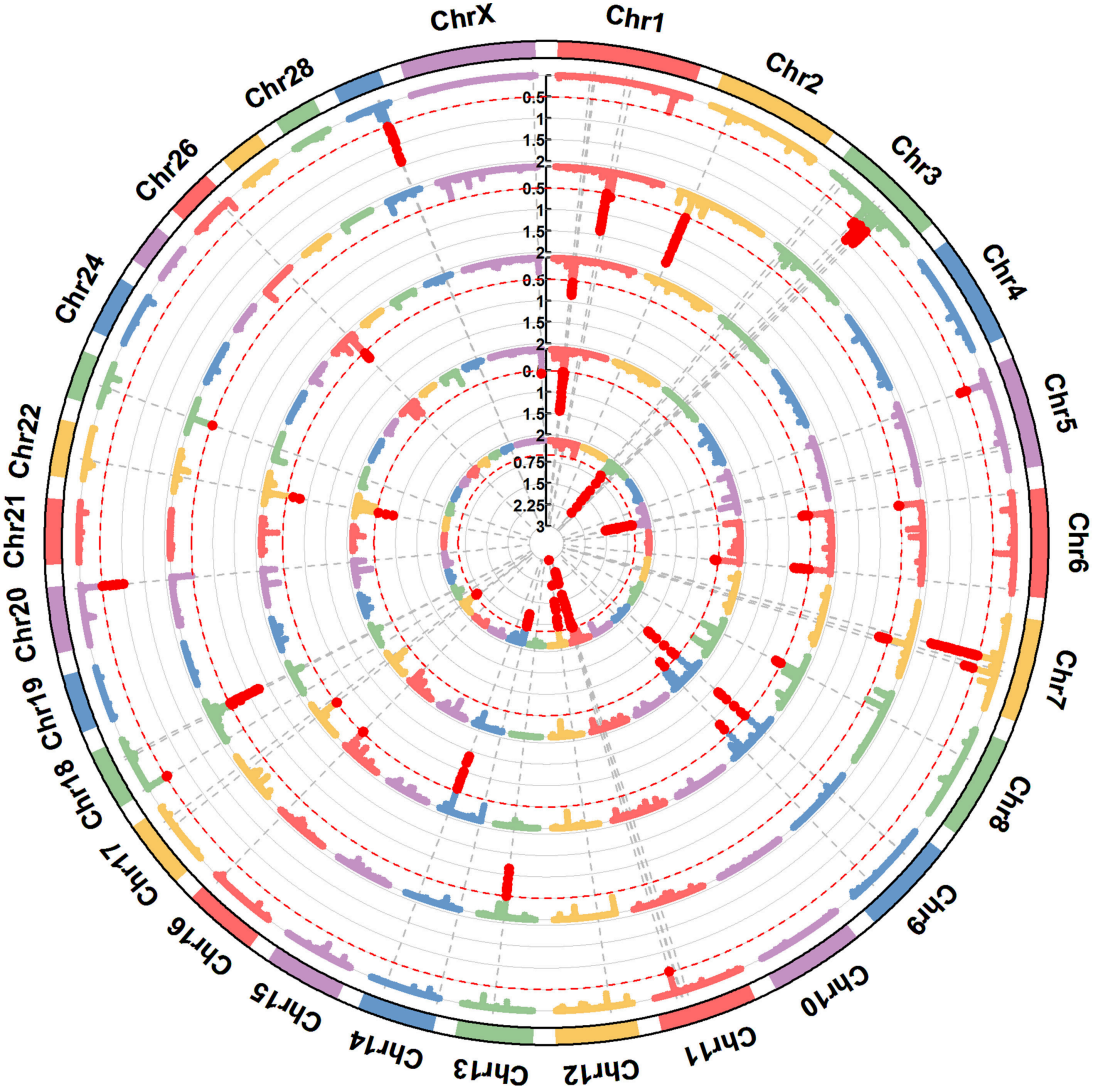
A circular-Manhattan plot for the proportion of genetic variance explained by the 5-SNP moving windows associated with the milk protein composition traits α-LA, β-LG, casein index, protein percentage, and protein yield. The horizontal line represents windows explaining no < 0.5% of the genomic variance. The five milk protein composition traits were plotted from inside to outside, respectively. A rectangular-Manhattan version of the plot is shown in the Supplementary Figures.

**Figure 3:**
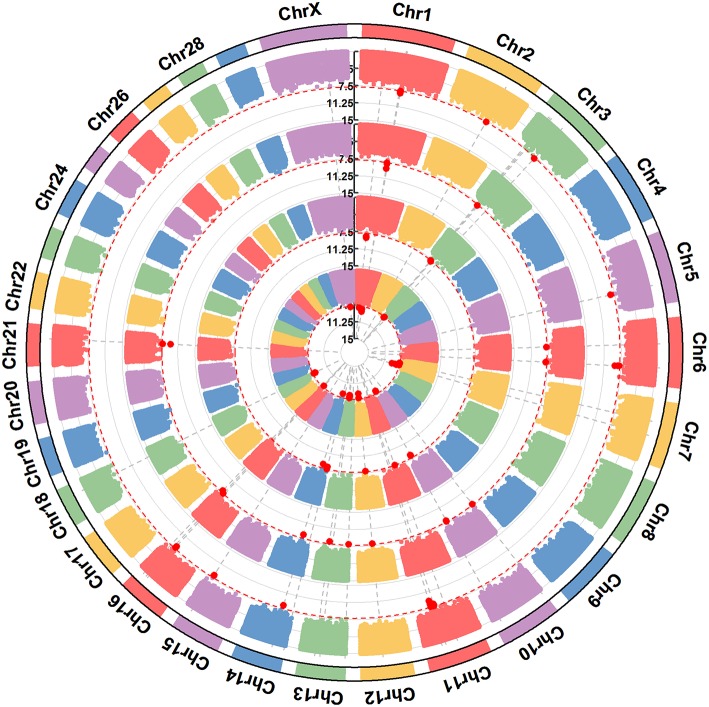
A circular-Manhattan plot for significance [–log_10_(*P*-values)] of the association of 586,304 SNPs based on analyses using ssGWAS located on 24 *Bos taurus* autosomes and the X chromosome with the milk protein composition traits α_s1_-CN, α_s2_-CN, β-CN, and κ-CN. The horizontal line represents a false discovery rate of 1%. The four milk protein composition traits were plotted from inside to outside, respectively. A rectangular-Manhattan version of the plot is shown in the Supplementary Figures.

**Figure 4:**
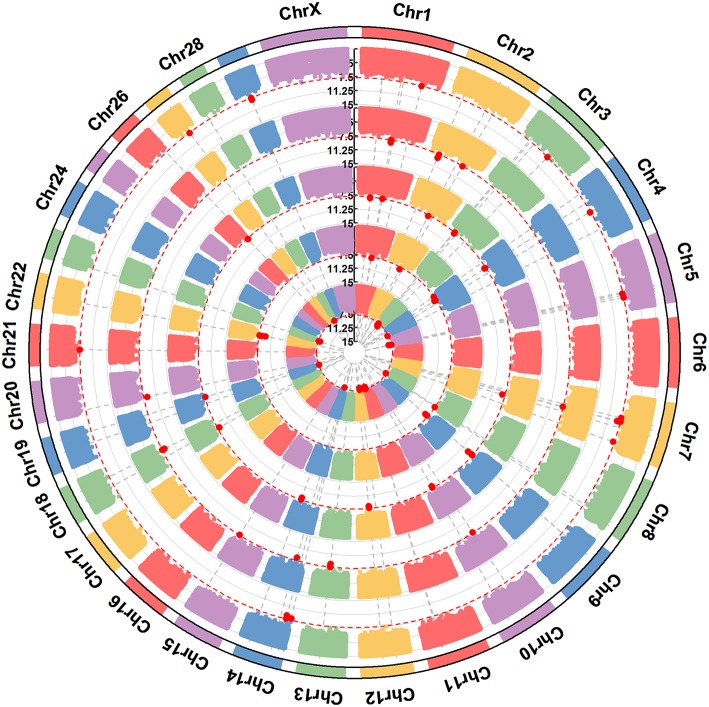
A circular-Manhattan plot for significance [–log_10_(*P*-values)] of the association of 586,304 SNPs based on analyses using ssGWAS located on 24 *Bos taurus* autosomes and the X chromosome with α_s1_-CN, α_s2_-CN, β-CN, and κ-CN. The horizontal line represents a false discovery rate of 1%. Five milk protein composition traits (α-LA, β-LG, casein index, protein percentage, and protein yield) were plotted from inside to outside, respectively. A rectangular-Manhattan version of the plot is shown in the Supplementary Figures.

In this study, we quantified milk protein composition using ELISA kits. The descriptive statistics of the phenotypes of milk protein composition traits are shown in Table 1. The mean concentrations of α_s1_-CN, α_s2_-CN, β-CN, κ-CN, α-LA, and β-LG were 35.45, 16.64, 31.23, 7.51, 2.25, and 6.93%, respectively. These results are similar to those previously obtained using capillary zone electrophoresis (CZE) (Schopen et al., 2011) and mid-infrared (MIR) spectra (Sanchez et al., 2017). The most abundant protein in milk was α_s1_-CN and the least abundant was α-LA. The allele correct rate was >96.0%, as determined through genotype imputation. A total of 178 informative windows of five adjacent SNPs were obtained for association with 586,304 SNPs using ssGWAS for all chromosomes and traits studied (Table 2 and Figures 1, 2). The main regions associated with milk protein composition traits were found on chromosomes BTA 1, 6, 7, 11, 13, 14, and 18. There were no significant associations on BTA 4, 19, 25, 27, or 28. A range of 11–31 significant windows was associated with all studied traits, and windows were located on 24 of the 29 bovine autosomes. A range of 1–31 windows per chromosome and 9–47% genetic variance were identified. Figures 3, 4 show the –log_10_(*P*-values) for association of the 586,304 SNPs obtained using ssGWAS for all the chromosomes and all the studied traits. Additional details relating to the SNPs associated with milk protein composition are shown in Table 2. We compared the results with the cattle QTL database (http://aaa.animalgenome.org/cgi-bin/QTLdb/BT/index) and found 118 reported QTLs related to milk protein composition traits. Relatively concentrated areas related to milk proteins were noted on chromosomes 6, 13, and 14. Twenty-eight QTLs related to four milk caseins (α_s1_-CN, α_s2_-CN, β-CN, and κ-CN) and protein yield were found on BTA 6, and 23 QTLs related to milk protein yield were found on BTA 13. Furthermore, 41 QTLs related to α_s2_-CN, α-LA, and milk protein yield were found on BTA 14 (Figure S19).

**Table 1:** Descriptive statistics of milk protein composition traits in a Chinese Holstein population.

**Traits^a^**	**No. cows**	**Mean**	**Standard deviation**	**Max**	**Min**
α_s1_-CN	614	35.45	17.46	72.63	1.96
α_s2_-CN	614	16.64	8.62	53.91	1.01
β-CN	614	31.23	10.31	69.59	2.24
κ-CN	614	7.51	1.69	23.48	0.43
α-LA	614	2.25	0.85	10.10	0.10
β-LG	614	6.93	3.67	48.64	0.18
Casein index^b^	614	90.51	7.01	99.16	49.25
Protein (%)	614	3.06	0.29	4.28	2.09
Protein (kg)	614	0.75	0.32	1.82	0.36

a *the six major milk proteins are expressed as a weight-proportion of the total protein fraction (wt/wt%)*.

b *casein index was calculated as [Σ casein/(Σ casein + Σ whey)] × 100*.

**Table 2:** Genomic regions associated with milk protein composition traits in a Chinese Holstein population.

**Traits**	**Window^a^**	**Chr**	**Start (bp)**	**End (bp)**	**Gene^b^**	**VE^c^ %**
α_s1_-CN	19,769	1	80,273,378	80,278,372	***SST***	0.53668
	19,774	1	80,279,371	80,285,703	–	0.97208
	19,779	1	80,286,424	80,295,590	–	1.18698
	162,874	6	1,169,171	1,184,671	–	0.65546
	162,879	6	1,185,355	1,196,443	–	0.80933
	162,885	6	1,200,729	1,213,176	–	0.70096
	172,237	6	38,611,254	38,618,402	*FAM184B,LAP3*	0.50646
	184,344	6	87,145,250	87,165,643	***CSN1S1***	0.74797
	208,632	7	64,546,663	64,556,472	***SLC36A3***	0.7675
	208,637	7	64,557,335	64,561,361	***SLC36A2***	0.84938
	208,642	7	64,561,888	64,565,585	***SLC36A2***	0.94219
	208,647	7	64,566,358	64,571,073	***SLC36A2***	0.98993
	251,700	9	28,174,804	28,184,358	–	0.80659
	324,134	11	98,031,589	9,804,0621	***GARNL3, FPGS***	0.76835
	358,993	13	46,208,034	46,227,765	–	0.63918
	358,998	13	46,239,050	46,266,967	–	0.8634
	359,150	13	46,826,742	46,832,055	***LARP4B, IDI1***	0.96543
	359,155	13	46,832,561	4,684,1024	***LARP4B, IDI1***	1.98644
	359,160	13	46,848,858	46,863,899	*DIP2C*	0.52083
	460,055	18	46,918,922	46,922,699	***WDR62**,TDRD12,LRFN3*	0.92899
	460,060	18	46,930,251	46,935,240	***WDR62**,SDHAF1,**CLIP3***	0.78047
	634,654	30	65,849,093	65,881,094	–	0.63984
α_s2_-CN	19,774	1	80,279,371	80,285,703	***SST***	0.61328
	19,779	1	80,286,424	80,295,590	–	0.7488
	162,879	6	1,185,355	1,196,443	–	0.50428
	208,642	7	64,561,888	64,565,585	***SLC36A2***	0.50889
	208,647	7	64,566,358	64,571,073	***SLC36A2***	0.52451
	251,700	9	28,174,804	28,184,358	–	0.5084
	358,998	13	46,239,050	46,266,967	–	0.51412
	359,150	13	46,826,742	46,832,055	***LARP4B, IDI1***	0.59555
	359,155	13	46,832,561	46,841,024	***LARP4B, IDI1***	1.2275
	366,525	14	1,880,378	1,923,292	*MROH1,HGH1,WDR97,**RPL8**,**DGAT1**,**HSF1**,BOP1*	9.58891
	366,530	14	1,943,598	1,962,021	*GPAA1,EXOSC4,**RPL8***	25.28515
	366,535	14	1,967,325	2,002,873	*GRINA,PARP10,PLEC*	5.98065
	460,055	18	46,918,922	46,922,699	***WDR62**,TDRD12,LRFN3,SDHAF1,**CLIP3***	0.5446
β-CN	19,774	1	80,279,371	80,285,703	***SST***	0.7799
	19,779	1	80,286,424	80,295,590	–	0.96602
	184,355	6	87,193,163	87,199,876	*HSTN,**CSN2***	1.08121
	208,632	7	64,546,663	64,556,472	*SLC36A3*	0.57655
	208,637	7	64,557,335	64,561,361	***SLC36A2***	0.64355
	208,642	7	64,561,888	64,565,585	***SLC36A2***	0.72545
	208,647	7	64,566,358	64,571,073	***SLC36A2***	0.76805
	282,267	10	42,294,691	42,301,905	–	0.51038
	324,134	11	98,031,589	9,804,0621	***GARNL3, FPGS***	1.05629
	359,150	13	46,826,742	46,832,055	***LARP4B, IDI1***	0.84256
	359,155	13	46,832,561	46,841,024	***LARP4B, IDI1***	1.73651
	359,258	13	47,256,972	47,267,747	*ZMYND11*	0.58935
	399,097	15	57,483,486	57,489,517	*MYO7A*	0.66981
	512,656	21	47,726,063	47,732,180	***SLC25A21***	0.82925
	512,661	21	47,732,701	47,736,349	***SLC25A21***	1.7035
	512,666	21	47,737,074	47,745,958	***SLC25A21***	2.03522
	512,672	21	47,747,607	47,753,773	***SLC25A21***	1.53975
	512,677	21	47,754,497	47,765,937	***SLC25A21***	0.71179
	512,682	21	47,769,663	47,789,155	***SLC25A21***	0.93541
	512,687	21	47,793,083	47,801,142	***SLC25A21***	0.96353
	512,692	21	47,803,810	47,817,554	***SLC25A21***	1.2754
	512,697	21	47,825,966	47,829,543	***SLC25A21***	1.67032
	512,702	21	47,830,117	47,836,983	***SLC25A21***	1.73322
	512,707	21	47,837,917	47,843,030	–	2.07561
	512,712	21	47,850,176	47,855,412	–	1.81429
κ-CN	20,211	1	82,294,481	82,305,519	–	1.10802
	20,243	1	82,431,764	82,437,922	***EHHADH***	0.54963
	175,209	6	49,471,344	49,479,118	–	0.54214
	184,357	6	87,194,926	87,202,745	*HSTN,**CSN1S1,CSN1S2,CSN3***	0.79865
	184,363	6	87,204,356	87,211,731		0.50442
	208,632	7	64,546,663	64,556,472	*SLC36A3*	0.67841
	208,637	7	64,557,335	64,561,361	***SLC36A2***	0.70792
	208,642	7	64,561,888	64,565,585	***SLC36A2***	0.72822
	208,647	7	64,566,358	64,571,073	***SLC36A2***	0.73794
	232,008	8	49,148,545	49,153,356	–	0.61036
	315,812	11	68,286,773	68,295,787	*SNRNP27*	0.67319
	315,817	11	68,297,079	68,318,091	*CAPN14,PCBP1*	1.06004
	315,823	11	68,321,826	68,338,425	*PCBP1*	0.50539
	317,833	11	75,577,993	75,582,470		0.84317
	317,838	11	75,583,309	75,588,583		2.70571
	460,055	18	46,918,922	46,922,699	***WDR62**,TDRD12,LRFN3,SDHAF1,**CLIP3***	0.50308
α-LA	75,815	3	10,232,899	10,236,850	–	0.56821
	77,849	3	17,901,972	17,915,013	*SMCP*	0.88169
	77,854	3	17,917,726	17,923,688	–	2.20539
	154,644	5	91,452,138	91,455,698	–	0.83477
	154,649	5	91,456,684	91,465,025	–	1.26306
	154,654	5	91,465,793	91,471,989	–	1.49212
	154,659	5	91,474,178	91,487,645	–	0.54008
	155,991	5	96,740,319	96,749,944	*GRIN2B*	1.06207
	315,887	11	68,597,446	68,610,076	*TIA1*	0.57891
	315,893	11	68,611,558	68,619,423	–	0.54673
	317,725	11	75,316,226	75,324,266	*KLHL29*	0.96811
	317,730	11	75,325,077	75,328,297	*KLHL29*	2.26328
	317,736	11	75,329,898	75,332,287	*KLHL29*	2.5407
	317,745	11	75,339,255	75,342,857	*KLHL29*	2.32541
	317,750	11	75,343,908	75,349,434	*KLHL29,ATAD2B*	2.47608
	317,756	11	75,350,440	75,354,084	*KLHL29*	1.25197
	318,213	11	76,936,751	76,942,341	–	0.74192
	318,218	11	76,943,628	76,955,286	–	0.54323
	333,946	12	27,090,788	27,095,189	–	2.99378
	333,951	12	27,097,379	27,109,296	–	1.37015
	366,526	14	1,892,559	1,943,598	*MROH1,HGH1,SHARPIN,CYC1,**RPL8,DGAT1,HSF1**,BOP1*	1.04997
	442,451	17	59,302,576	59,320,664	*TAOK3*	0.59173
β-LG	10,258	1	41,702,974	41,708,148	***ARL6***	0.62806
	10,263	1	41,711,818	41,717,537	–	1.20529
	10,268	1	41,718,143	41,724,240	–	1.41982
	10,273	1	41,731,422	41,735,786	–	0.58327
	10,738	1	43,612,247	43,615,867	*COL8A1*	0.67198
	10,743	1	43,619,121	43,622,066	*COL8A1*	0.57725
	191,785	6	114,167,098	114,173,397	–	0.59503
	251,699	9	28,174,206	28,176,701	–	1.38642
	263,801	9	79,157,545	79,181,555	–	0.68722
	525,321	22	23,303,686	23,317,156	***IL5RA**,CRBN*	0.89145
	643,206	30	146,085,436	146,090,954		0.57121
Casein index	10,263	1	41,711,818	41,717,537	***ARL6***	0.73896
	10,268	1	41,718,143	41,724,240	–	0.83967
	162,874	6	1,169,171	1,184,671	–	0.50837
	162,879	6	1,185,355	1,196,443	–	0.65363
	162,885	6	1,200,729	1,213,176	–	0.5992
	191,785	6	114,167,098	114,173,397	–	0.8451
	228,942	8	34,914,343	3,492,0024	–	0.50943
	228,947	8	34,920,926	34,931,510	–	0.64162
	251,699	9	28,174,206	28,176,701	–	1.34694
	263,801	9	79,157,545	79,181,555	–	0.80926
	380,353	14	65,190,322	65,210,369	–	1.35986
	380,361	14	65,234,975	65,243,654	–	0.55361
	424,730	16	74,232,901	74,247,827	*KCNH1*	0.52587
	439,916	17	49,155,508	49,162,447	–	0.51494
	525,321	22	23,303,686	23,317,156	***IL5RA**,CRBN*	0.8046
	588,158	26	43,246,598	43,254,505	***ACADSB***	0.59819
	588,163	26	43,258,359	43,263,853	*HMX3*	0.68211
	588,168	26	43,280,115	43,298,983	*BUB3*	0.57729
Protein percentage	88,095	3	58,699,144	58,704,486	–	0.57847
	90,093	3	67,116,998	67,135,360	*MIGA1*	0.91725
	91,191	3	72,687,317	72,735,466	–	0.52308
	91,968	3	76,691,451	76,701,563	–	0.75867
	91,975	3	76,705,320	76,714,787	–	0.86154
	91,981	3	76,718,920	76,728,381	–	0.84187
	91,987	3	76,750,610	76,758,792	–	0.55404
	137,245	5	15,966,349	15,996,910	–	0.60869
	205,886	7	53,866,175	53,872,425	–	0.85908
	205,891	7	53,873,542	53,879,198	–	1.16389
	205,896	7	53,879,949	53,884,717	–	1.2126
	205,901	7	53,885,243	53,890,802	***PCDHB4***	1.23202
	205,906	7	53,893,497	53,910,675	***PCDHB4***	1.19168
	205,913	7	53,922,097	53,932,408	***–***	0.66998
	205,918	7	53,932,886	53,940,442	***PCDHB6***	0.55591
	205,927	7	53,947,702	53,952,520	–	0.69142
	205,933	7	53,959,180	53,965,425	*TAF7*	0.61338
	205,941	7	53,972,700	53,977,311	***PCDHB7***	0.75775
	205,946	7	53,978,290	53,984,004	***PCDHB16***	1.22971
	205,951	7	53,986,955	53,993,012	***PCDHB16***	1.55122
	210,377	7	70399534	70,402,362	–	0.71595
	366,523	14	1,861,799	1,911,696	*MROH1,HGH1,WDR97,**RPL8,DGAT1,HSF1**,BOP1*	0.90380
	366,529	14	1,923,292	1,954,317	*CYC1,GPAA1,EXOSC4,MAF1*	0.68963
	447,290	18	1,678,695	1,681,656	***COG4**,SF3B3*	0.50785
	447,295	18	1,682,755	1,690,385	***FUK***	0.50909
	499,941	20	68,427,642	68,435,666	–	0.65724
	499,946	20	68,442,658	68,449,729	–	0.95117
	625,513	29	39,932,584	39,935,454	*HRASLS5*	1.32356
	625,866	29	41,322,154	41,328,249	–	0.65351
	625,871	29	41,330,454	41,343,749	–	0.89201
	625,877	29	41,346,361	41,363,919	–	0.83036
	625,883	29	41,366,511	41,373,237	–	0.73769
Protein yield (kg)	19,769	1	80,273,378	80,278,372	***SST***	0.59587
	19,774	1	80,279,371	80,285,703	–	1.09238
	19,779	1	80,286,424	80,295,590	–	1.38891
	21,033	1	87,273,950	87,286,594	–	0.58122
	45,141	2	23,570,814	23,579,859	–	0.87377
	45,146	2	23,581,521	23,584,485	–	1.66946
	45,153	2	23,595,626	23,605,094	*MAP3K20*	1.31014
	45,158	2	23,605,919	23,621,394	–	0.57615
	162,878	6	1,184,671	1,195,799	–	0.53473
	208,632	7	64,546,663	64,556,472	*SLC36A3*	0.61681
	208,637	7	64,557,335	64,561,361	***SLC36A2***	0.66098
	208,642	7	64,561,888	64,565,585	***SLC36A2***	0.70393
	208,647	7	64,566,358	64,571,073	***SLC36A2***	0.72533
	359,150	13	46,826,742	46,832,055	***LARP4B, IDI1***	0.50278
	359,155	13	46,832,561	46,841,024	***LARP4B, IDI1***	1.14033
	459,118	18	43,379,174	43,385,147	*TDRD12*	0.76653
	460,050	18	46,914,865	46,918,136	*TDRD12*	0.51543
	460,055	18	46,918,922	46,922,699	***WDR62**,TDRD12,LRFN3,SDHAF1,**CLIP3***	1.26331
	460,060	18	46,930,251	46,935,240	***WDR62**,TDRD12,LRFN3,SDHAF1,**CLIP3***	1.19527
	460,065	18	46,936,044	46,940,696	***WDR62***	0.92135
	460,071	18	46,943,334	46,950,099	***WDR62***	0.70592
	460,080	18	46,955,527	46,958,825	*OVOL3,POLR2I,TBCB,CAPNS1*	0.70892
	460,085	18	46,960,023	46,967,172	*OVOL3,POLR3I,TBCB,COX7A1*	0.55181
	541,509	23	235,290,93	23,541,032	–	0.52547

a *window that consists of five adjacent SNPs*.

b *positional/putative candidate gene*.

c *genomic variance absorbed by 5-SNP moving windows obtained using single-step genomic-BLUP*.

*The meaning of the bold values is that pointing out these genes is promising candidate genes affecting milk protein concentration*.

### Genome-Wide Association Study

Several informative windows on BTA 1 and 6 showed highly significant associations with five major milk proteins (α_s1_-CN, α_s2_-CN, β-CN, κ-CN, and β-LG), casein index, protein yield, and protein percentage. We identified a continuous genomic region on BTA 7 associated with α_s1_-CN, α_s2_-CN, β-CN, κ-CN, protein yield, and protein percentage. Additionally, BTA 11, 13, and 14 each had significant associations with four studied traits (BTA 11: α_s1_-CN, β-CN, κ-CN, and α-LA; BTA 13: α_s1_-CN, α_s2_-CN, β-CN, and protein yield; and BTA 14: α_s2_-CN, α-LA, casein index, and protein percentage). A number of windows of BTA 18 also had associations with αs1-CN, α_s2_-CN, κ-CN, protein yield, and protein percentage.

In total, we detected 22, 13, 25, 16, 22, 11, 18, 30, and 24 informative windows for α_s1_-CN, α_s2_-CN, β-CN, κ-CN, α-LA, 11 β-LG, casein index, protein percentage, and protein yield, respectively. Four windows (64.54–64.57 Mbp) explained 3.55% of the genetic variance in total and the most significant SNP (BovineHD0700018734) associated with α_s1_-CN was located in a 64.5-Mbp region on BTA 7 within the *SLC36A2* gene. An important window from 87.14 to 87.16 Mbp was located on BTA 6 within the *CSN1S1* gene, which is a major gene affecting α_s1_-CN in dairy cattle. The three most informative windows explaining 40.85% of the genetic variance associated with α_s2_-CN were located within a region from 18.80 to 20.02 Mbp on BTA 14. In this region, the SNP BovineHD1400000256 showing the strongest association was located 0.9 Mbp from the *DGAT1* gene, which influences milk composition in dairy cattle. Twelve of 25 informative windows explaining 17.29% of the genetic variance for β-CN were clustered on BTA 21 in a region from 47.72 to 47.85 Mbp that contains the *SLC25A21* gene. A significant SNP, BovineHD2100013628, located at 87.19 Mbp on BTA 6 was located 0.01 Mbp from the *CSN2* gene, which is a major gene influencing α_s2_-CN. Four windows associated with κ-CN were located within a region from 64.54 to 64.57 Mbp on BTA 7 containing the *SLC36A2* gene. A significant SNP, BovineHD0600023887, within an informative window from 87.19 to 87.21 Mbp on BTA 6 was located 0.21 Mbp from the *CSN3* gene. A region containing 10 windows explaining 14.23% of the genetic variance from 68.59 to 76.95 Mbp on BTA 11 was strongly associated with α-LA. The most informative window for β-LG was identified within a region containing six windows from 41.70 to 43.62 Mbp on BTA 1.

The two most informative windows associated with casein index were located within a region from 65.19 to 65.24 Mbp on BTA 14. The most significant associations with protein percentage and protein yield were clustered on BTA 7 within a 16.50-Mbp segment that included 13 windows (53.86–70.40 Mbp) that explained 12.44% of the genetic variance and a 3.70-Mbp segment that included eight windows (43.37–46.96 Mbp) that explained 6.63% of the genetic variance.

#### Candidate Genes and Functional Analyses

**Figure 5:**
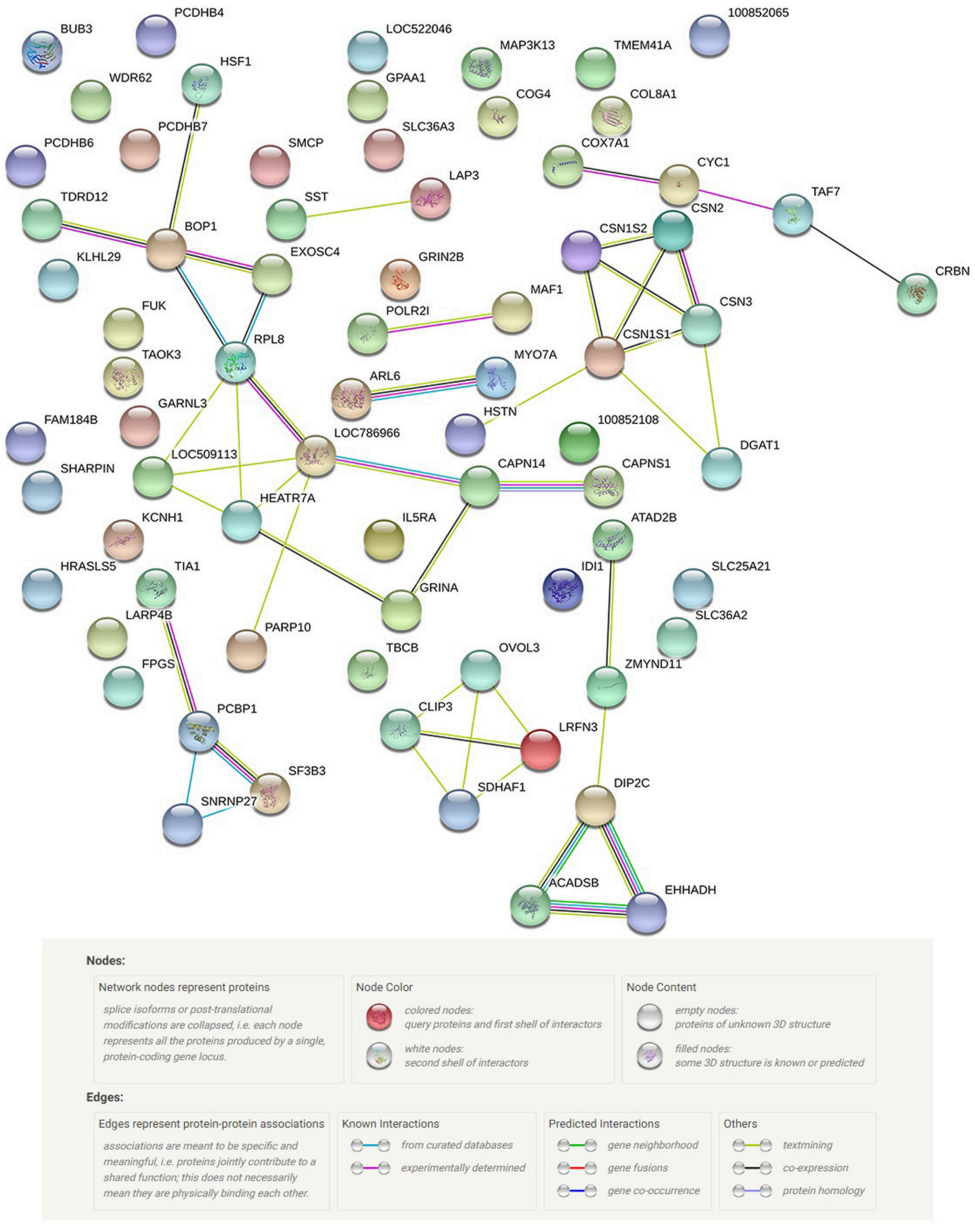
Protein network of the 62 most probable candidate genes detected, according to STRING v10.0 action view.

A total of 62 functional genes were located in or close to windows that explained no < 0.5% of the genomic variance. PPI and GO enrichment analyses were performed for the 62 most plausible candidate genes. The interaction network of proteins encoded by these genes was more extensive and significant than expected (46 edges identified; PPI enrichment *P* = 2.7 e^−14^; Figure 5). We also identified significantly enriched GO terms (false discovery rate < 0.05) for four biological processes and 12 cellular components with four to 24 of these genes for milk protein composition traits (Table 3). On the basis of the functional annotation results, PPI findings, and the biological processes shown in the DAVID analysis, we finally identified 27 prospective candidate genes for milk composition traits with biological functions, including amino acid metabolism, amino acid transport, protein metabolism, and Golgi transport and subsequent modification: *ARL6, SST, EHHADH* (BTA 1), *CSN1S1, CSN1S2, CSN2, CSN3, LAP3* (BTA 6), *PCDHB4, PCDHB6, PCDHB7, PCDHB16, SLC36A2* (BTA 7), *GALNT14, FPGS* (BTA 11), *LARP4B, IDI1* (BTA 13), *RPL8, HSF1, DGAT1* (BTA 14), *COG4, FUK, WDR62, CLIP3* (BTA 18), *SLC25A21* (BTA 21), *IL5RA* (BTA 22), and *ACADSB* (BTA 26).

**Table 3:** Gene Ontology (GO) functional enrichment with false discovery rate (FDR) < 0.05.

	**Pathway ID**	**Description**	**Gene count**	**FDR**
Biological Process	GO.1903494	Response to dehydroepiandrosterone	4	2.16E-06
	GO.1903496	Response to 11-deoxycorticosterone	4	2.16E-06
	GO.0032570	Response to progesterone	4	2.25E-05
	GO.0032355	Response to estradiol	4	0.000345
Cellular Component	GO.0005796	Golgi lumen	4	2.45E-06
	GO.0070013	Intracellular organelle lumen	16	4.22E-05
	GO.0044446	Intracellular organelle part	23	0.000109
	GO.0044428	Nuclear part	12	0.00617
	GO.0043227	Membrane-bounded organelle	24	0.0103
	GO.0044444	Cytoplasmic part	19	0.0143
	GO.0043231	Intracellular membrane-bounded organelle	22	0.0227
	GO.0043229	Intracellular organelle	23	0.0238
	GO.0005737	Cytoplasm	23	0.0247
	GO.0031981	Nuclear lumen	10	0.0247
	GO.0043226	Organelle	23	0.0397
	GO.0044431	Golgi apparatus part	5	0.0483

## Discussion

In this study, we quantified milk protein composition using ELISA kits, and we conducted a single-step GWAS using imputed 777 K chips of 614 Chinese Holstein cows. A total of 178 significant windows for all studied milk composition traits were detected, among which some windows are located within known QTL regions on BTA 1, 6, 14, and 11 (Schopen et al., 2011; Sanchez et al., 2017). However, in the present study, we found no associations between regions on BTA 6 and αs2-CN or between regions on BTA 11 and β-LG, probably due to the different dairy populations that were selected. Several regions were found to be located within or close to genes that are known to have functions related to milk composition. In addition, 25 promising candidate genes for milk protein composition were identified.

### Chromosomes Containing Novel Candidate Genes for Milk Composition Traits

On chromosome BTA 1, a total of 21 windows were associated with α_s1_-CN, α_s2_-CN, β-CN, κ-CN, β-LG, casein index, and protein yield. Windows associated with β-LG and casein index were located 0.23 Mbp from the *ARL6* gene, which encodes ADP ribosylation factor like GTPase 6 and is involved in membrane protein trafficking. *ARL6* has been implicated in mammary gland cell membrane trafficking and microtubule dynamics (Kahn et al., 2005; Rao et al., 2012). The somatostatin (*SST*) and 3-hydroxyacyl coenzyme A dehydrogenase (*EHHADH*) genes are located in a region of BTA 1 (80.2–82.4 Mbp) that is associated with α_S1_-CN, α_S2_-CN, β-CN, and protein yield. Somatostatin (somatotropin release inhibiting factor, SRIF) is an endogenous cyclic polypeptide and abundant neuropeptide with two biologically active forms that exert a wide range of physiological effects on neurotransmission, secretion, and cell proliferation. Somatostatin is also potentially associated with lactation as a signaling molecule (Lupoli et al., 2001). The protein encoded by *EHHADH* is a bifunctional enzyme and one of the four enzymes of the peroxisomal beta-oxidation pathway. This gene is highly inducible via peroxisome proliferator-activated receptor α (PPARα) activation and has a key influence on milk composition traits (Houten et al., 2012).

We detected 30 windows between 53 and 64 Mbp on BTA 7 that were associated with α_s1_-CN, α_s2_-CN, β-CN, κ-CN, protein yield, and protein percentage. Twelve adjacent windows (53.86–53.99 Mbp) were strongly associated with protein percentage and contain multiple genes (*PCDHB4, PCDHB6, PCDHB7*, and *PCDHB16*), including the protocadherin beta gene cluster, which is critically involved in the establishment and function of specific cell–cell neural connections in humans (Tan et al., 2010). Moreover, two common contiguous windows within *SLC36A2* (64.56–64.57 Mbp) were associated with six milk protein composition traits (α_s1_-CN, α_s2_-CN, β-CN, κ-CN, protein yield, and protein percentage). *SLC36A2* plays a key role in amino acid transport across the plasma membrane as well as the transport of glucose and other sugars, bile salts and organic acids, metal ions, and amine compounds (Edwards et al., 2018), and may therefore have pleiotropic effects for several milk protein composition traits.

On BTA 11, we identified a region of 17 windows from 68 to 98 Mbp that was associated with α_s1_-CN, β-CN, κ-CN, and α-LA. There was a strong association between α-LA and a region of five windows (68.28–68.61 Mbp) located 0.1 Mbp from the *GALNT14* (polypeptide *N-*acetylgalactosaminyl transferase 14) gene, a member of the polypeptide *N*-acetylgalactosaminyl transferase (ppGalNAc-Ts) protein family. These enzymes catalyze the transfer of *N*-acetyl-d-galactosamine to the hydroxyl groups on serines and threonines of target peptides. The encoded protein participates in protein metabolism (Wang et al., 2003). Therefore, *GALNT14* has potential effects on α-LA. Additionally, a segment at 98 Mbp associated with α_s1_-CN and β-CN was located 0.4 Mbp from the *FPGS* gene. The folylpolyglutamate synthase enzyme encoded by *FPGS* plays a central role in establishing and maintaining both cytosolic and mitochondrial folylpolyglutamate concentrations. Further, *FPGS* is involved in several key metabolic pathways, including those associated with folate biosynthesis and the metabolism of vitamins and cofactors. Therefore, *FPGS* potentially serves as a bridge between metabolism and synthesis for α_s1_-CN and β-CN (Oppeneer et al., 2012).

We detected a total of 13 windows on BTA 13 that were associated with α_s1_-CN, α_s2_-CN, β-CN, and protein yield. Two of these windows (46.82–46.84 Mbp) were located 0.40 Mbp and 1.7 Mbp from the *LARP4B* and *IDI1* genes, respectively*. LARP4B* encodes a member of an evolutionarily conserved protein family and is implicated in RNA metabolism and translation. This protein family includes five sub-families: one genuine La protein and four La-related protein (LARP) sub-families. *LARP4B* may stimulate amino acid transport as a cytoplasmic protein (Mattijssen and Maraia, 2016). IDI1 plays a key role in the metabolism of nutrients in the liver and is involved in milk protein synthesis. Therefore, both *LARP4B* and *IDI1* are promising candidate genes for milk protein composition traits (Shi et al., 2018).

On BTA 18, a total of 14 windows were associated with α_s1_-CN, α_s2_-CN, κ-CN, protein yield, and protein percentage. The *COG4* and *FUK* genes were noted in a region containing two adjacent windows (16.78–16.90 Mbp) that were associated with protein percentage. *COG4* (component of oligomeric Golgi complex 4) is a protein-coding gene that is involved in the structure and function of the Golgi apparatus, whereas *FUK* (fucokinase) is involved in protein metabolism and transport to the Golgi and subsequent modification. Thus, *COG4* and *FUK* may play key roles in the transport of milk proteins. The *WDR62* (WD repeat domain 62) gene was also located in a region that included nine windows (43.37–46.96 Mbp) with significant associations with α_s1_-CN, α_s2_-CN, κ-CN, protein yield, and protein percentage. *WRD62* encodes a c-Jun N-terminal kinase scaffold protein. Scaffold proteins such as WRD62 simultaneously associate with various components of the MAPK signal pathway and play a crucial role in signal transmission and MAPK regulation. The MAPK pathway regulates cellular proliferation and differentiation, in part by controlling protein translation machinery (Sciascia et al., 2013). Therefore, *WDR62* may play a significant role in milk protein synthesis. Additionally, the *CLIP3* (CAP-Gly domain-containing linker protein 3) gene, which encodes a member of the cytoplasmic linker proteins of 170 family, was located 0.28 Mbp from this region. Members of this protein family contain a cytoskeleton-associated protein glycine-rich domain and mediate the interaction of microtubules with cellular organelles.

Finally, 11 contiguous windows associated with β-CN were located from 47.72 to 47.85 Mbp on BTA 21 containing the *SLC25A21* (solute carrier family 25 member 21) gene, which encodes a protein that participates in amino acid metabolism (Scarcia et al., 2017). On BTA 22, we detected an informative window (23.30–23.31 Mbp) that was significantly associated with β-LG and casein index and included the *IL5RA* (interleukin 5 receptor subunit alpha) gene. As a novel milk protein gene, *IL5RA* activates multiple downstream Jak-STAT signaling pathways and is involved in proteasome-mediated ubiquitin-dependent protein catabolism. On BTA 26, three adjacent windows (43.24–43.29 Mbp) that were significantly associated with casein index were located proximal to the *ACADSB* (acyl-CoA dehydrogenase short/branched chain) gene, the encoded protein of which is involved branched-chain amino acid catabolism (Liu et al., 2013).

### Chromosomes Containing Known Candidate Genes for Milk Composition Traits

We identified 16 windows on BTA 6 (87.19–87.21 Mbp) that were associated with α_s1_-CN, α_s2_-CN, β-CN, κ-CN, β-LG, casein index, and protein yield. This segment included the casein gene cluster containing the *CSN1S1, CSN1S2, CSN2*, and *CSN3* genes, which encode α_s1_, α_s2_, β, and κ casein, respectively. The casein gene cluster has a strong influence on casein synthesis in bovine milk, and polymorphisms in this region have significant effects on milk protein composition and cheese-making abilities (Grosclaude, 1988; Grisart et al., 2002). Additionally, a window associated with α_s1_-CN located at 38.61 Mbp was 0.11 Mbp from the *LAP3* (leucine aminopeptidase 3) gene. As a known gene affecting milk production traits, *LAP3* is involved in arginine and proline metabolism and affects protein maturation and degradation (Zheng et al., 2011), thereby potentially affecting casein synthesis.

A 2-Mbp region of BTA 14 (18.61–20.02 Mbp) containing six windows was associated with α_s1_-CN, β-CN, κ-CN, and α-LA. In this region, we identified the SNP BovineHD1400007026 as being most significantly associated with αS1-CN, αS2-CN, β-CN, κ-CN, α-LA, and protein yield. Several genes were identified in this region, including *DGAT1*, which has major effects on milk protein, milk fat content, and mineral composition in bovine milk (Schennink et al., 2007; Bovenhuis et al., 2016). The *RPL8* gene, located 2.7 Mbp from this region, probably plays an important role in the transcriptional regulation of *DGAT1* and may exert significant effects on milk production traits in dairy cattle (Jiang et al., 2010, 2014). Therefore, both *DGAT1* and *RPL8* are important candidate genes for milk protein composition traits. Additionally, the *HSF1* gene, located 0.6 Mbp from this region, is involved in ERK signaling and the cellular response to heat stress. The protein encoded by *HSF1* is rapidly induced in response to temperature stress and binds heat shock promoter elements. *HSF1* has a significant effect on milk production mediated by a lysine-232/alanine polymorphism in the bovine *DGAT1* gene (Winter et al., 2002). Therefore, *HSF1* may have indirect effects on milk proteins such as α_s1_-CN, β-CN, κ-CN, and α-LA.

## Conclusions

In the present study, we identified a total of 178 genomic windows and 194 SNPs on 24 bovine autosomes that were significantly associated with milk protein composition or protein percentage, including six genomic regions on chromosomes BTA 1, 6, 11, 13, 14, and 18. Within these regions, we identified the following 27 candidate genes for milk composition traits: *ARL6, SST, EHHADH, CSN1S1, CSN1S2, CSN2, CSN3, LAP3, PCDHB4, PCDHB6, PCDHB7, PCDHB16, SLC36A2, GALNT14, FPGS, LARP4B, IDI1, RPL8, HSF1, DGAT1, COG4, FUK, WDR62, CLIP3, SLC25A21, IL5RA*, and *ACADSB*. The findings of this study provide an important foundation for future fine-mapping studies to more precisely elucidate the mutations affecting milk protein composition traits in dairy cattle. Future studies should establish causative links between candidate variants and milk protein phenotypes using functional analyses.

## Data Availability

The genotype and phenotype data of the samples used in the present study are available from the FigShare Repository: https://figshare.com/s/206a2bcbf0cb0e4c2564

## Ethics Statement

Milk samples were collected from farms that periodically undergo quarantine inspections. The entire collection process was performed in strict accordance with a protocol approved by the AnimalWelfare Committee of China Agricultural University (permit number: DK996).

## Author Contributions

SZ conceived and designed the study, and revised the manuscript. CZ performed the phenotype collection, sample collection, data analysis, and drafted the manuscript. CL participated in the experimental design and drafted the manuscript. SL, WC, and SS participated in sample collection. QZ participated in data interpretation and manuscript revision. All authors have read and approved the final manuscript.

### Conflict of Interest Statement

The authors declare that the research was conducted in the absence of any commercial or financial relationships that could be construed as a potential conflict of interest.

## Supplementary Material

Table S1 Single-nucleotide polymorphisms showing the most significant association with six major milk proteins, casein index, protein percentage, and protein yield.

Figure S1 Manhattan plot for the proportion of genetic variance explained by the 5-SNP moving windows associated with α_s1−_CN.

Figure S2 Manhattan plot for the proportion of genetic variance explained by the 5-SNP moving windows associated with α_s2_-CN.

Figure S3 Manhattan plot for the proportion of genetic variance explained by the 5-SNP moving windows associated with β-CN.

Figure S4 Manhattan plot for the proportion of genetic variance explained by the 5-SNP moving windows associated with κ-CN.

Figure S5 Manhattan plot for the proportion of genetic variance explained by the 5-SNP moving windows associated with α-LA.

Figure S6 Manhattan plot for the proportion of genetic variance explained by the 5-SNP moving windows associated with β-LG.

Figure S7 Manhattan plot for the proportion of genetic variance explained by the 5-SNP moving windows associated with casein index.

Figure S8 Manhattan plot for the proportion of genetic variance explained by the 5-SNP moving windows associated with protein percentage.

Figure S9 Manhattan plot for the proportion of genetic variance explained by the 5-SNP moving windows associated with protein yield.

Figure S10 Manhattan plot for the –log_10_ (*P*-value) of single SNP associated with α_s1−_CN.

Figure S11 Manhattan plot for the –log_10_ (*P-*value) of single SNP associated with α_s2_-CN.

Figure S12 Manhattan plot for the –log_10_ (*P-*value) of single SNP associated with β-CN.

Figure S13 Manhattan plot for the –log_10_ (*P-*value) of single SNP associated with κ-CN.

Figure S14 Manhattan plot for the –log_10_ (*P-*value) of single SNP associated with α-LA.

Figure S15 Manhattan plot for the –log_10_ (*P-*value) of single SNP associated with β-LG.

Figure S16 Manhattan plot for the –log_10_ (*P-*value) of single SNP associated with casein index.

Figure S17 Manhattan plot for the –log_10_ (*P-*value) of single SNP associated with protein percentage.

Figure S18 Manhattan plot for the –log_10_ (*P-*value) of single SNP associated with protein yield.

Figure S19 Genetic map for 118 QTL related to milk protein composition.
