# Supplementary material for: Genome-Wide Association Study for Milk Protein Composition Traits in a Chinese Holstein Population Using a Single-Step Approach
Source: Front Genet. 2019 Feb 19;10:72. doi: 10.3389/fgene.2019.00072 (PMC6389681; doi:10.3389/fgene.2019.00072)
Supplement: Figure S1 — Manhattan plot for the proportion of genetic variance explained by the 5-SNP moving windows associated with αs1−CN. [file Data_Sheet_1.pdf]

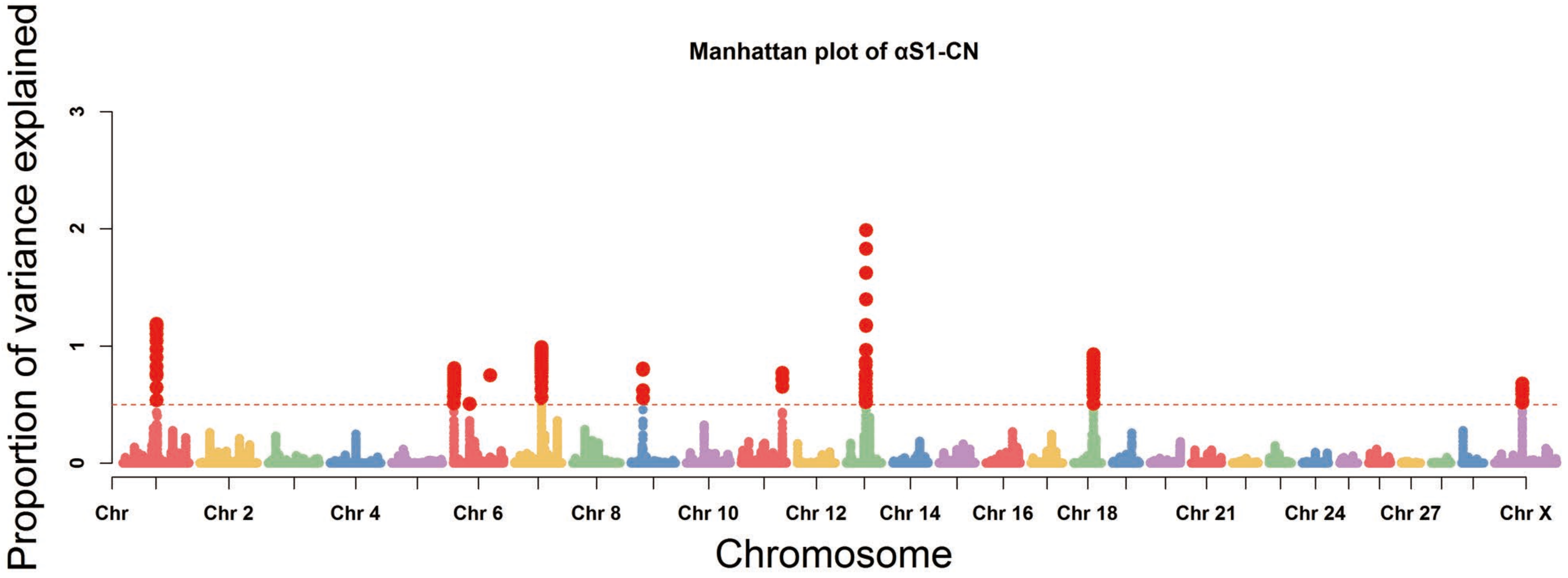

Fig. S1. Manhattan plot for the proportion of genetic variance explained by the 5-SNP moving windows associated with αs1-CN

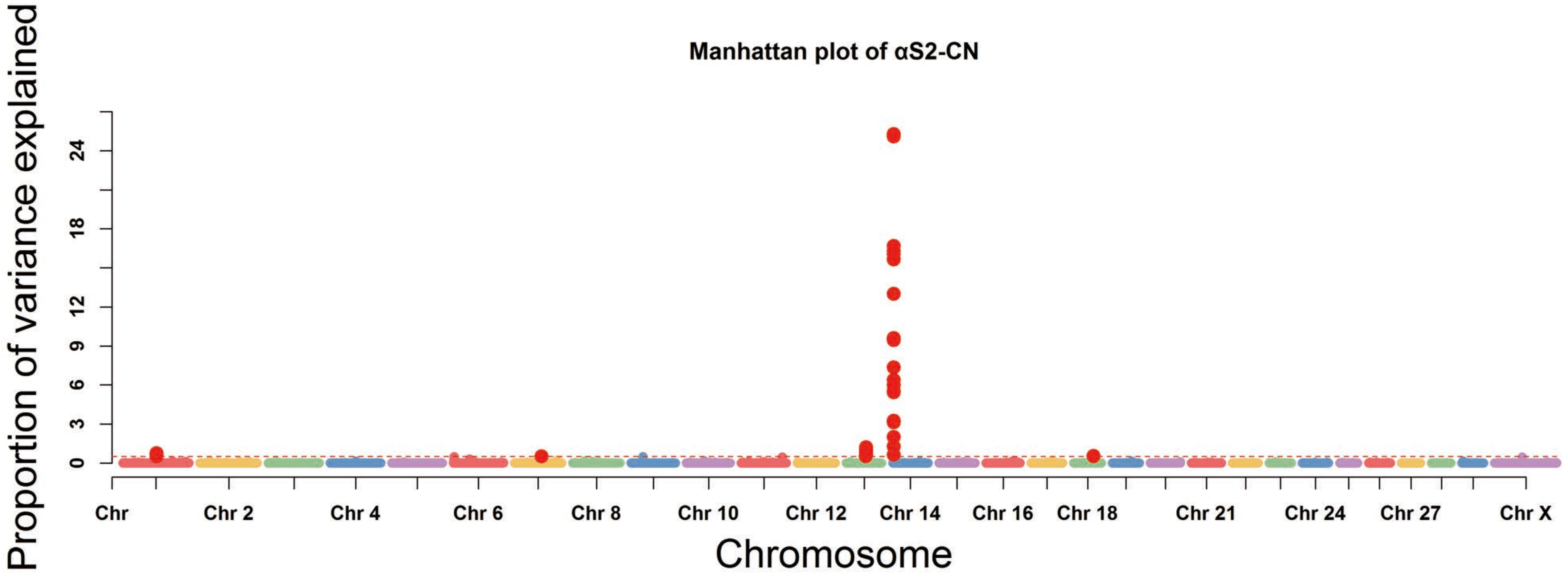

Fig. S2. Manhattan plot for the proportion of genetic variance explained by the 5-SNP moving windows associated with αs2-CN

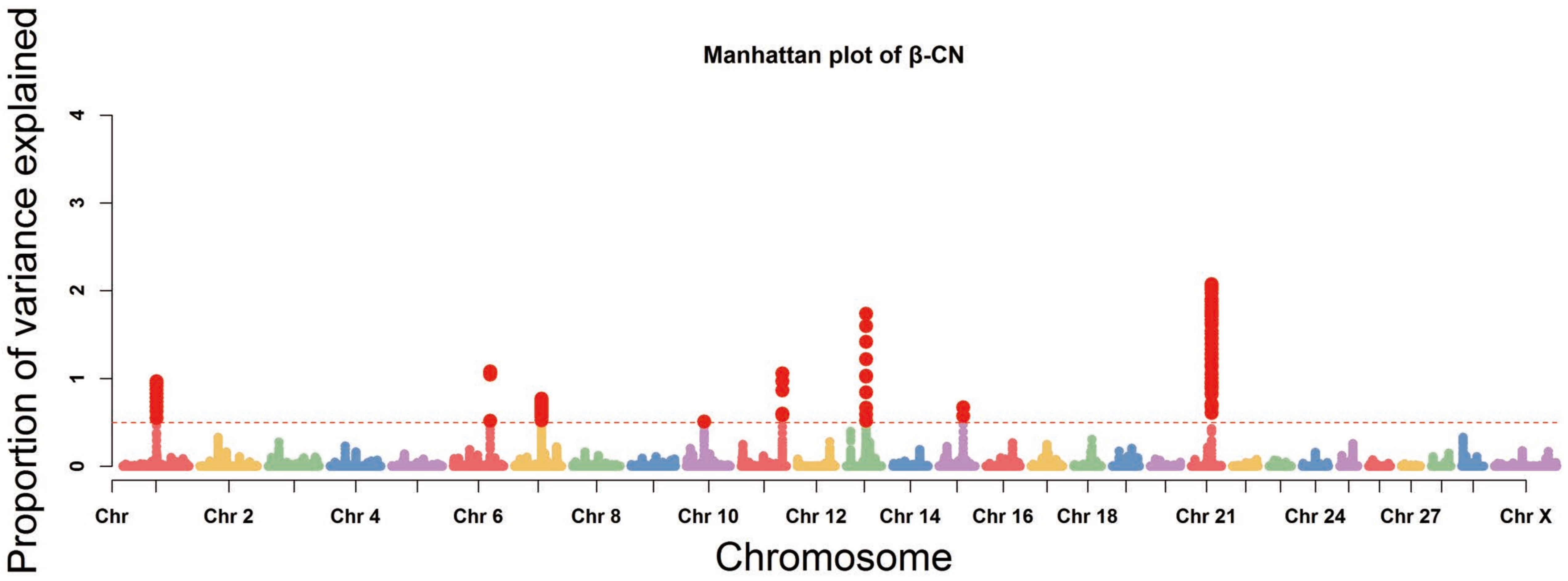

Fig. S3. Manhattan plot for the proportion of genetic variance explained by the 5-SNP moving windows associated with β-CN

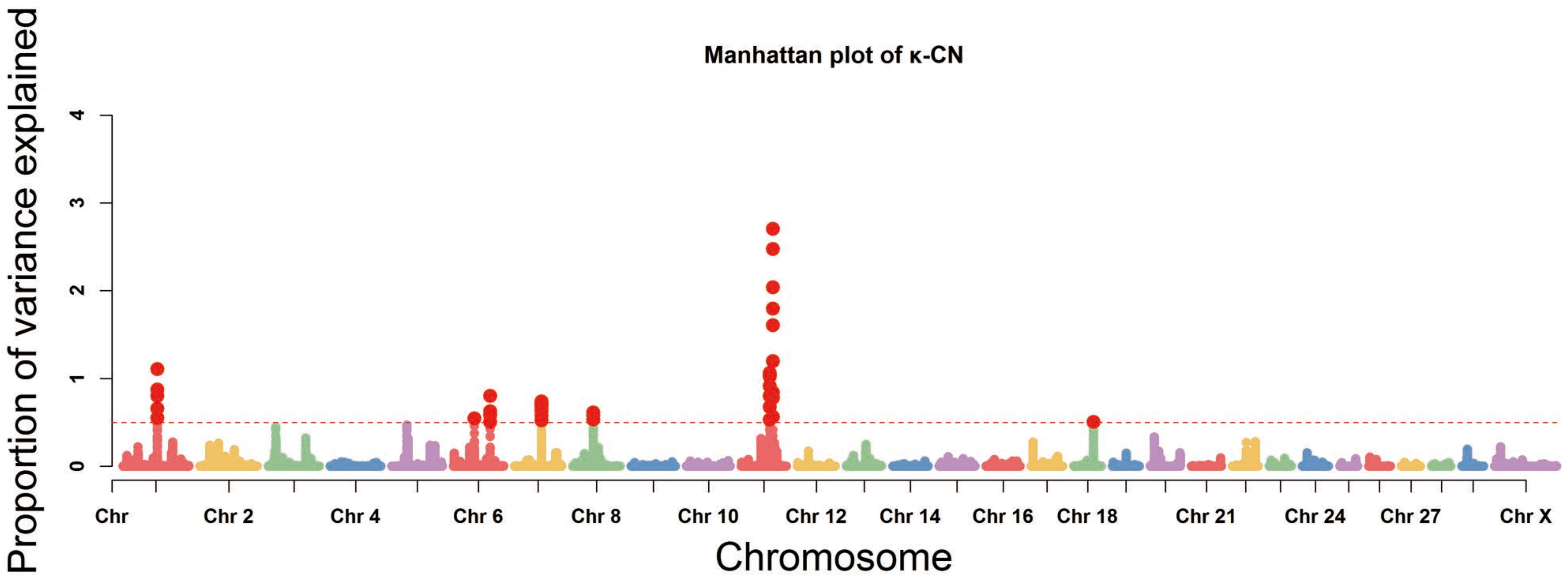

Fig. S4. Manhattan plot for the proportion of genetic variance explained by the 5-SNP moving windows associated with κ-CN

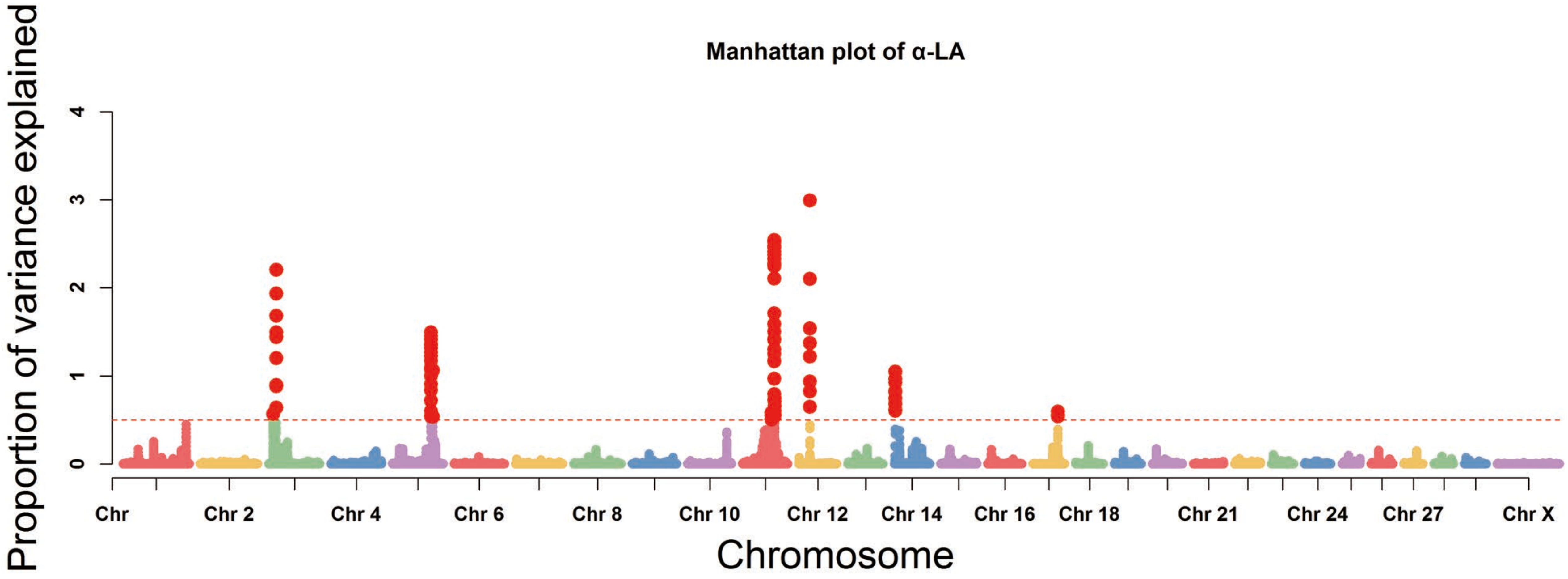

Fig. S5. Manhattan plot for the proportion of genetic variance explained by the 5-SNP moving windows associated with α-LA

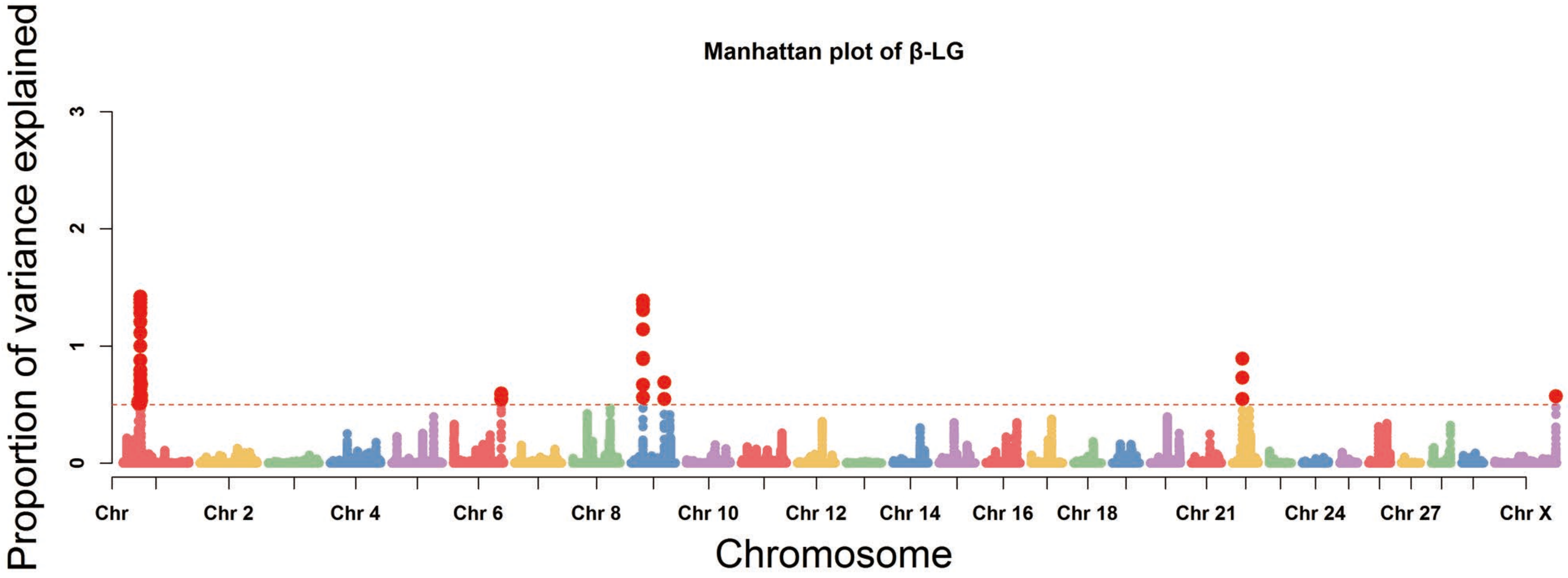

Fig. S6. Manhattan plot for the proportion of genetic variance explained by the 5-SNP moving windows associated with β-LG

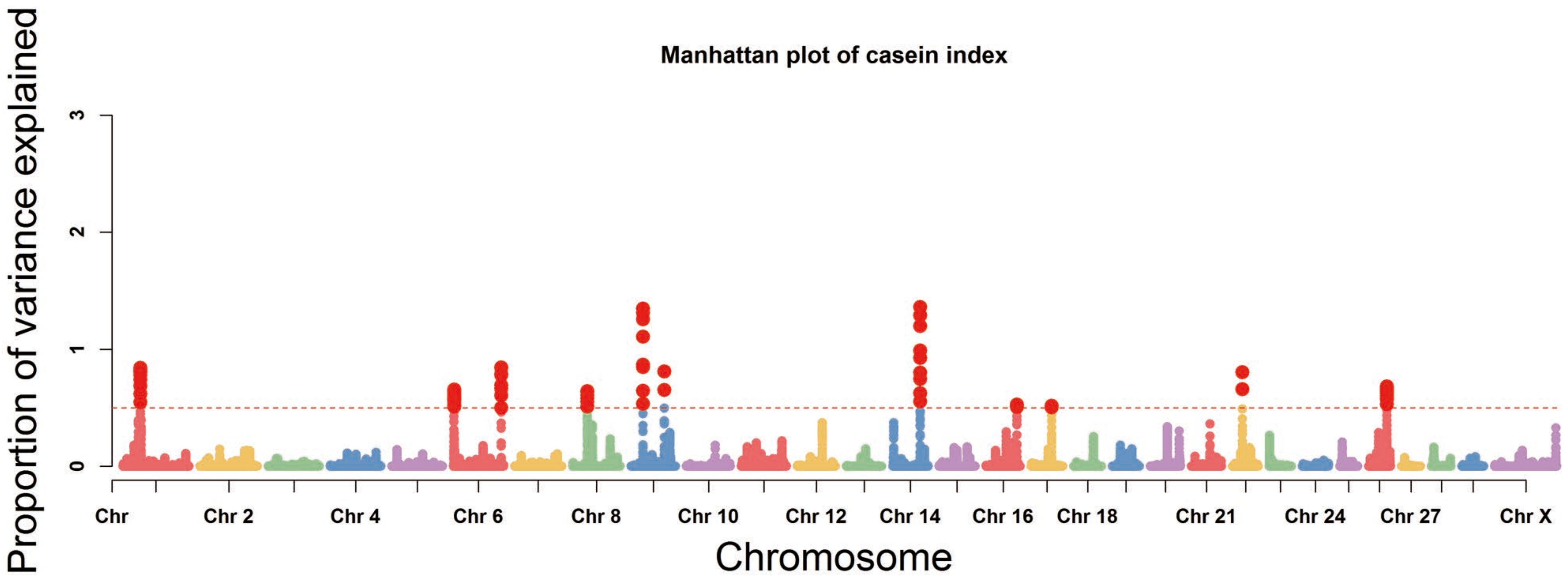

Fig. S7. Manhattan plot for the proportion of genetic variance explained by the 5-SNP moving windows associated with casein index

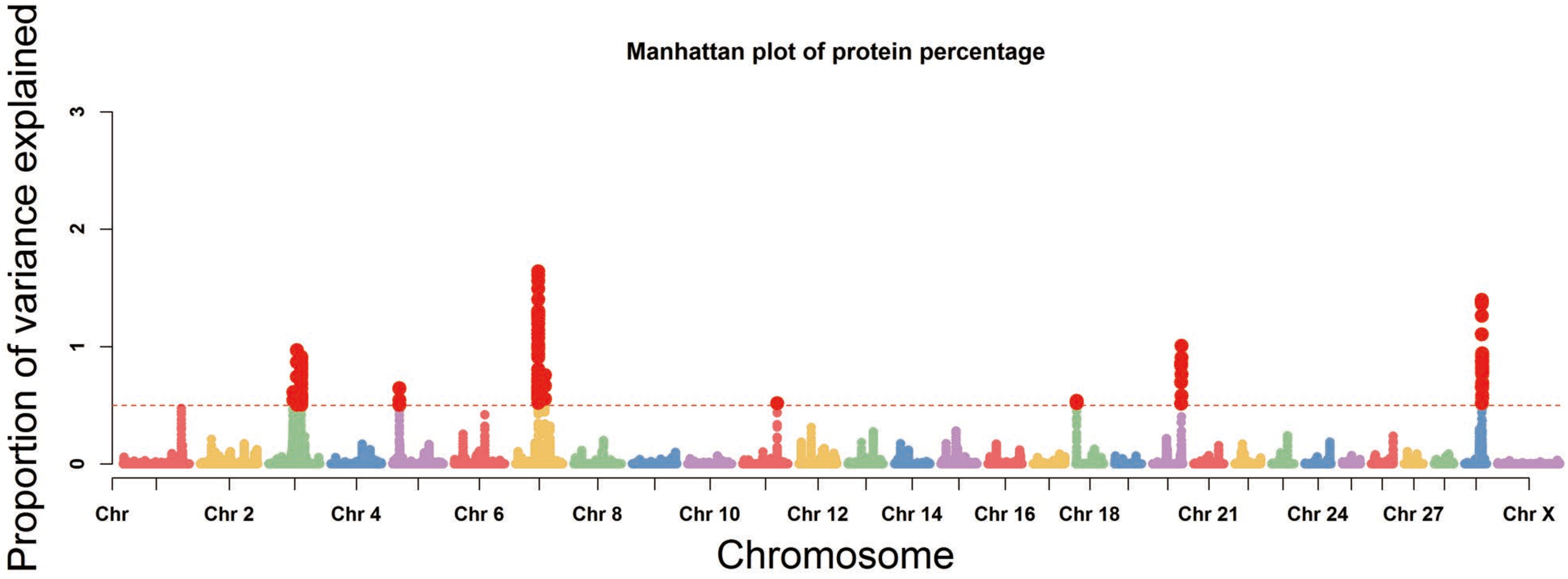

Fig. S8. Manhattan plot for the proportion of genetic variance explained by the 5-SNP moving windows associated with protein percentage

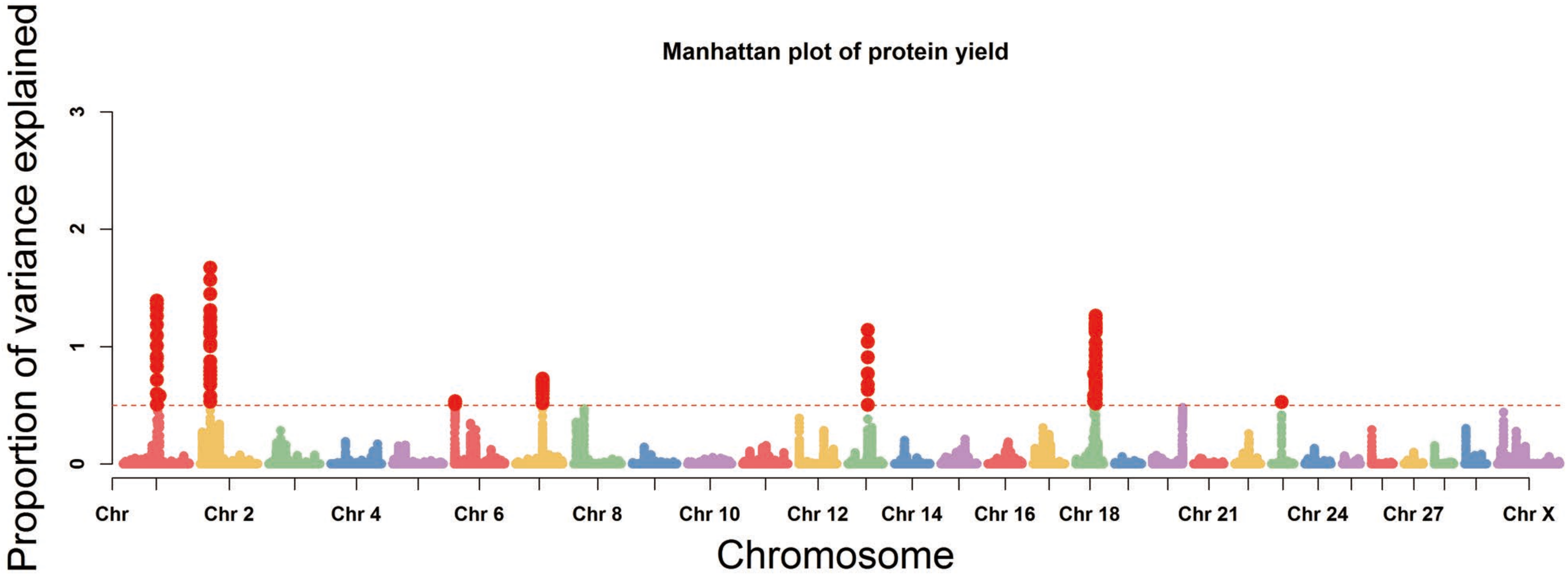

Fig. S9. Manhattan plot for the proportion of genetic variance explained by the 5-SNP moving windows associated with protein yield
