# Supplementary material for: Genome-Wide Association Study for Milk Protein Composition Traits in a Chinese Holstein Population Using a Single-Step Approach
Source: Front Genet. 2019 Feb 19;10:72. doi: 10.3389/fgene.2019.00072 (PMC6389681; doi:10.3389/fgene.2019.00072)
Supplement: Figure S10 — Manhattan plot for the –log10 (P-value) of single SNP associated with αs1−CN. [file Data_Sheet_2.pdf]

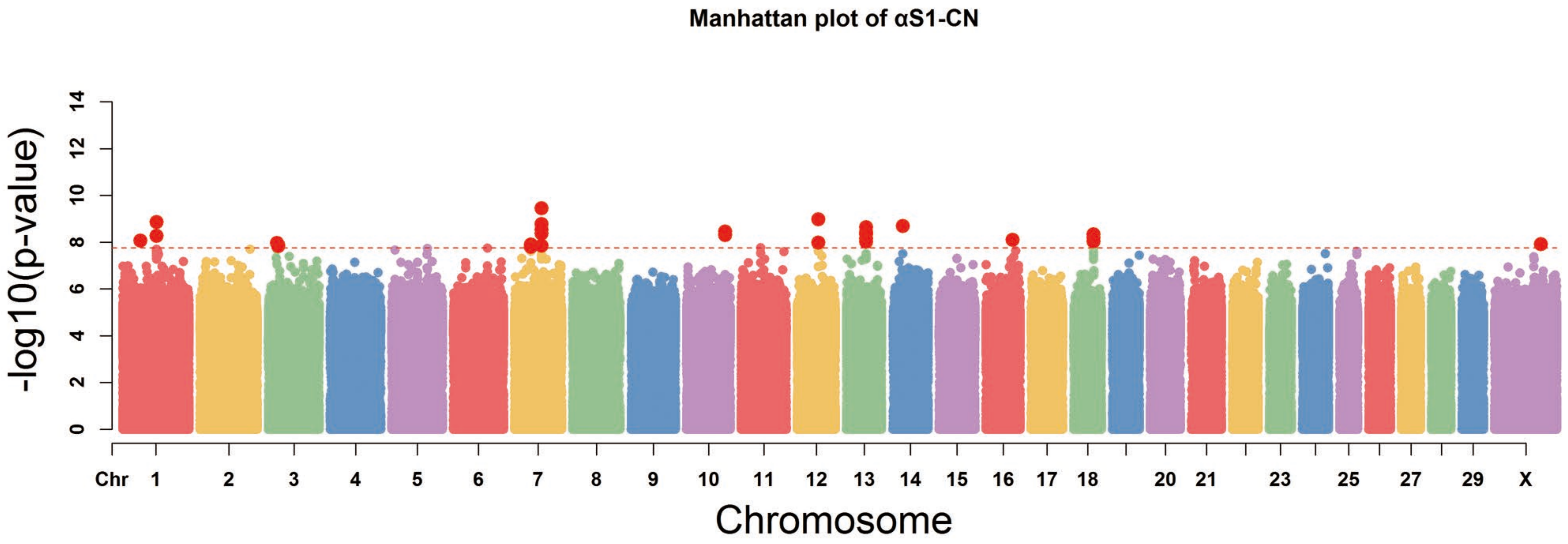

Fig. S10. Manhattan plot for the –log10 (P-value) of single SNP associated with αs1-CN

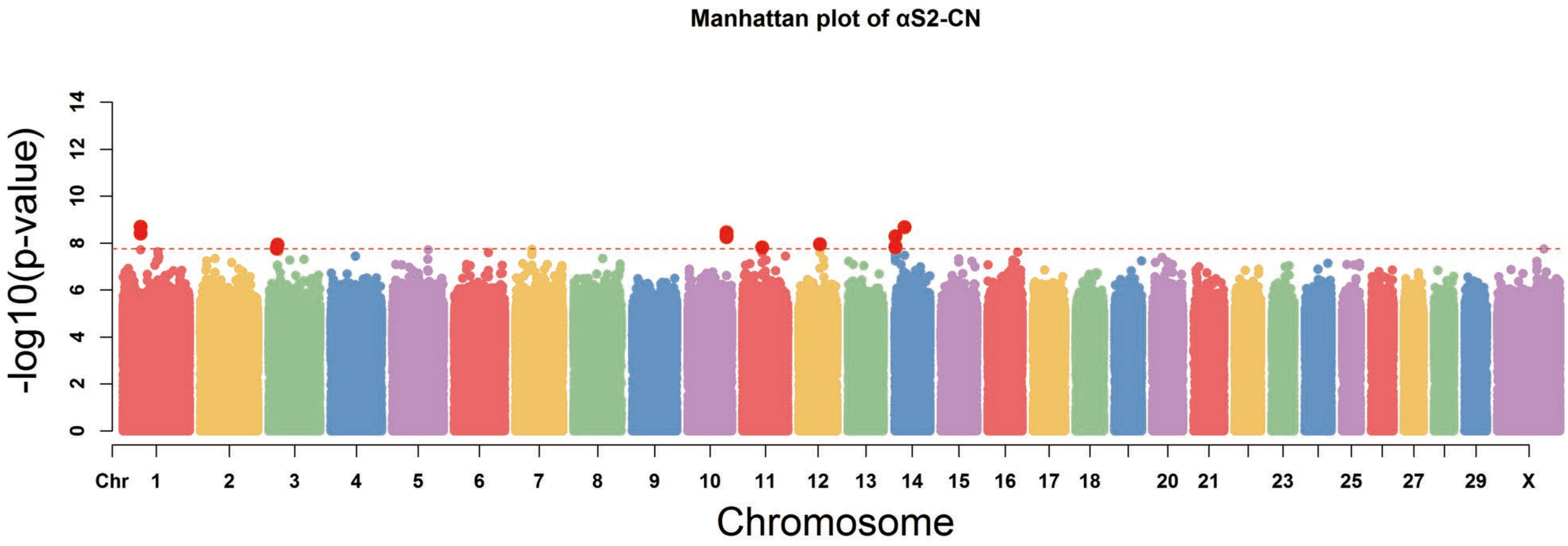

Fig. S11. Manhattan plot for the –log10 (P-value) of single SNP associated with αs2-CN

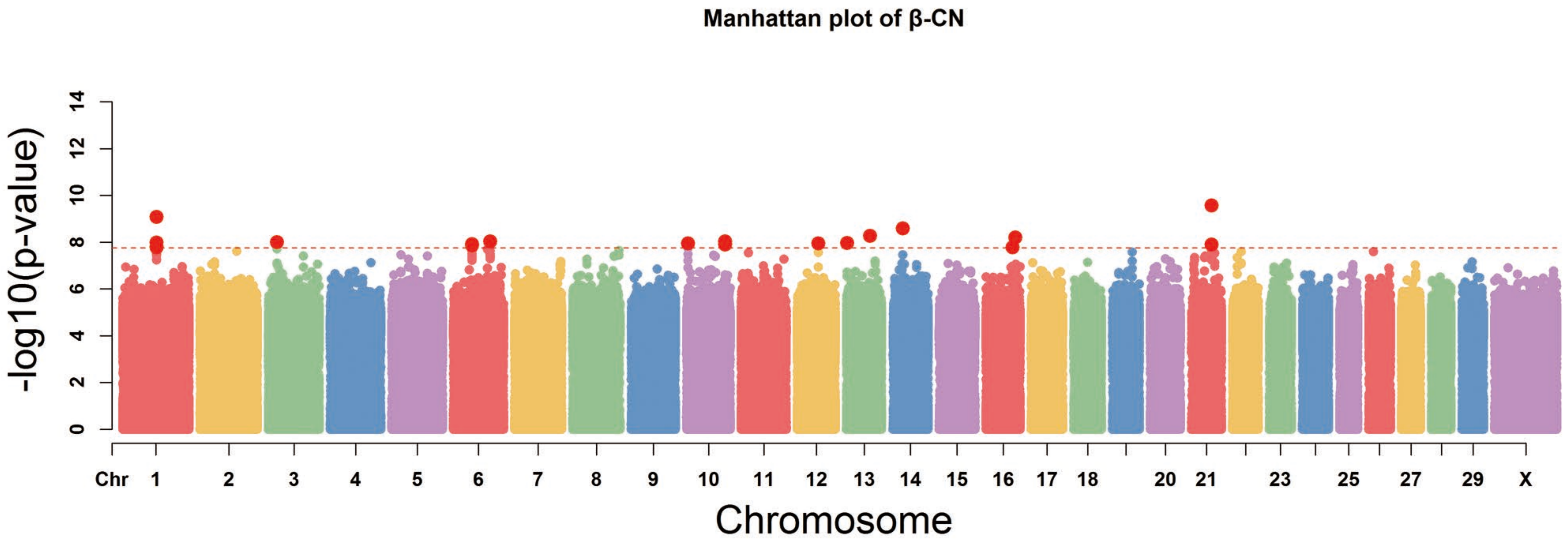

Fig. S12. Manhattan plot for the –log10 (P-value) of single SNP associated with β-CN

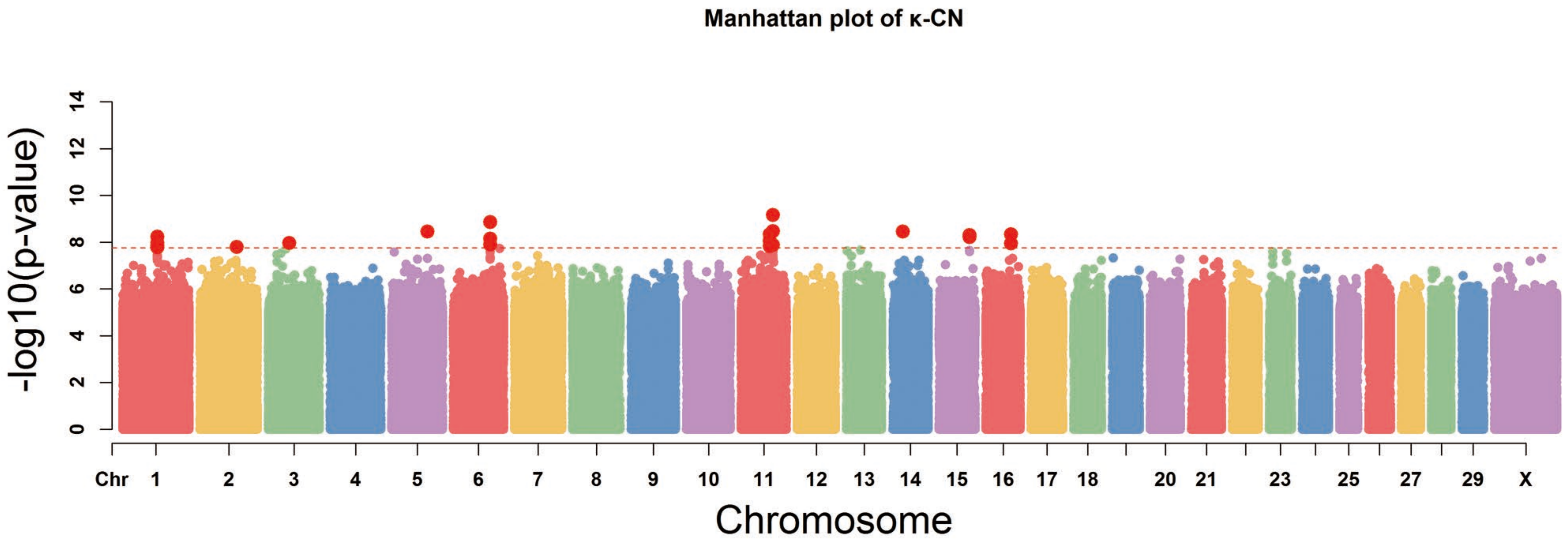

Fig. S13. Manhattan plot for the –log10 (P-value) of single SNP associated with κ-CN

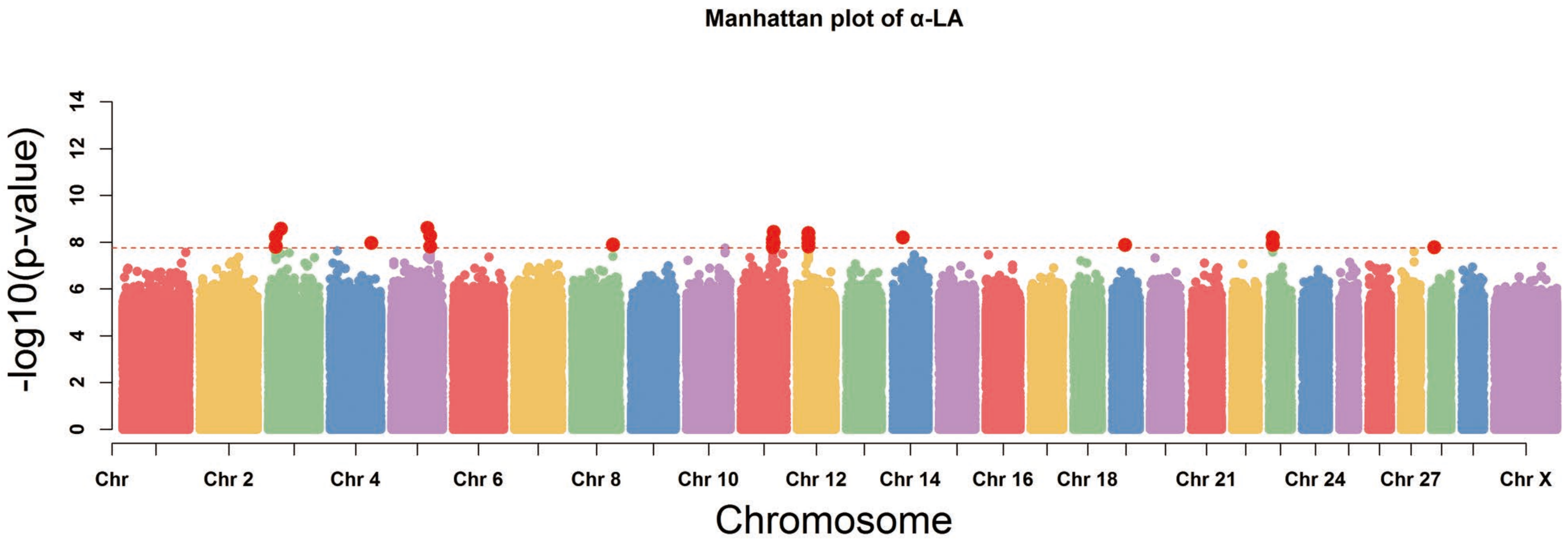

Fig. S14. Manhattan plot for the –log10 (P-value) of single SNP associated with α-LA

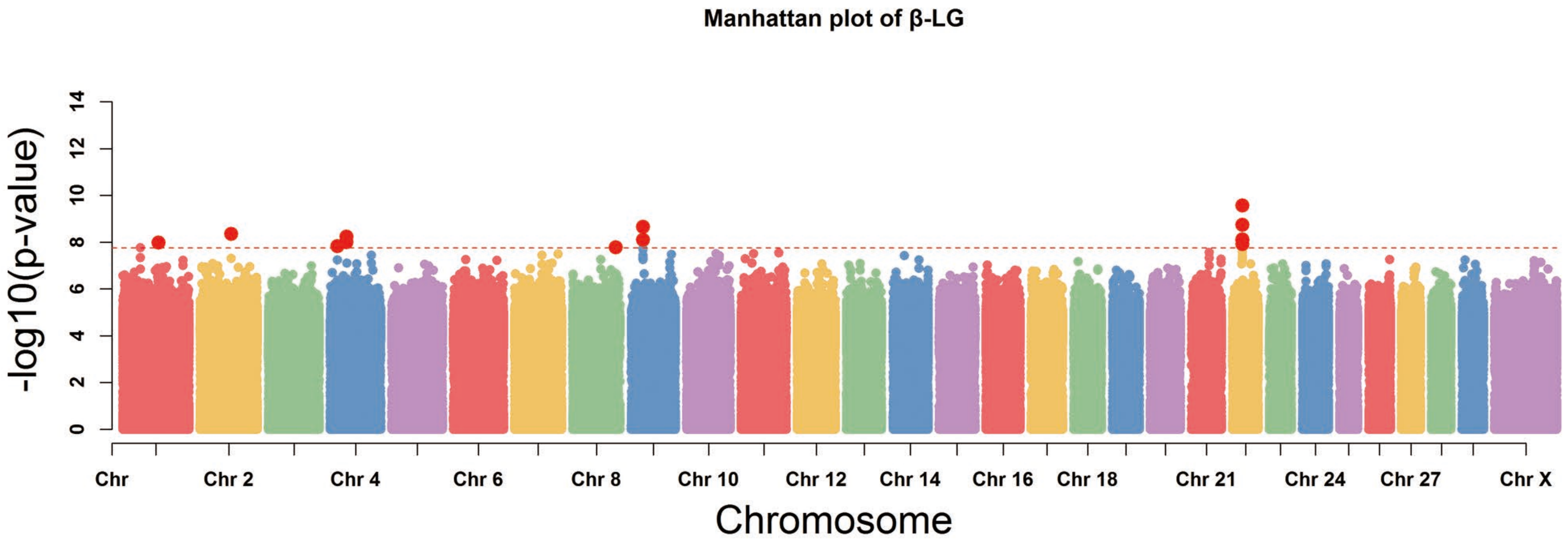

Fig. S15. Manhattan plot for the –log10 (P-value) of single SNP associated with β-LG

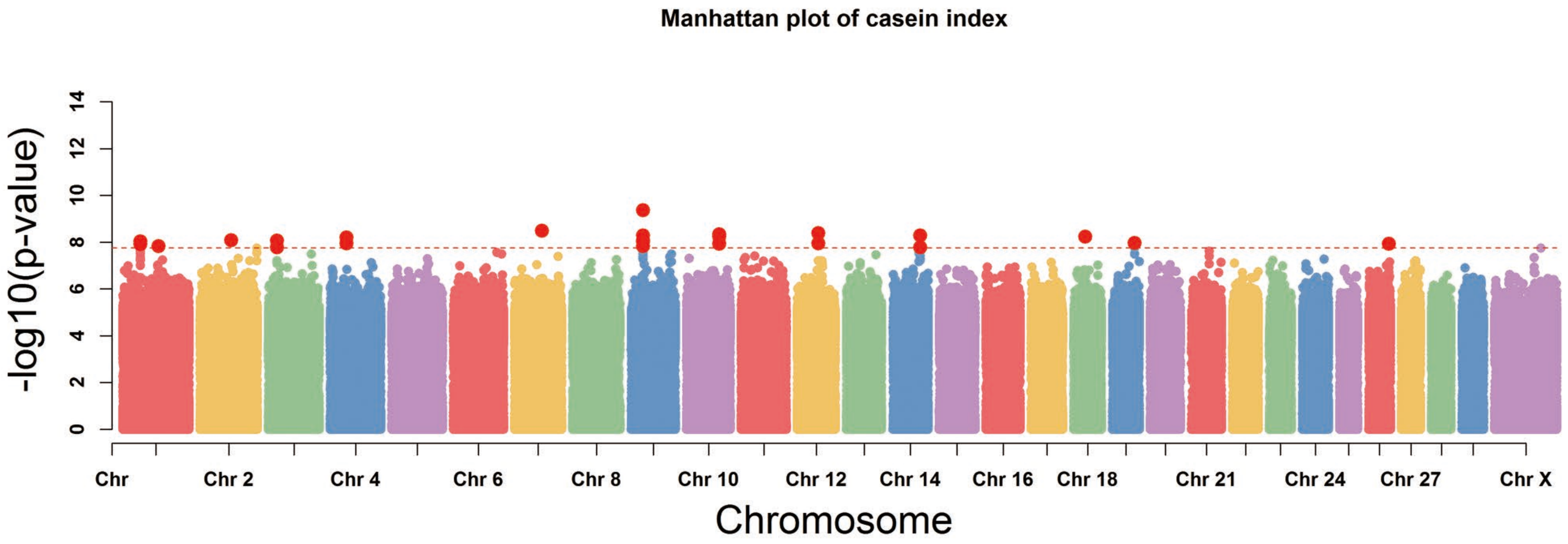

Fig. S16. Manhattan plot for the –log10 (P-value) of single SNP associated with casein index

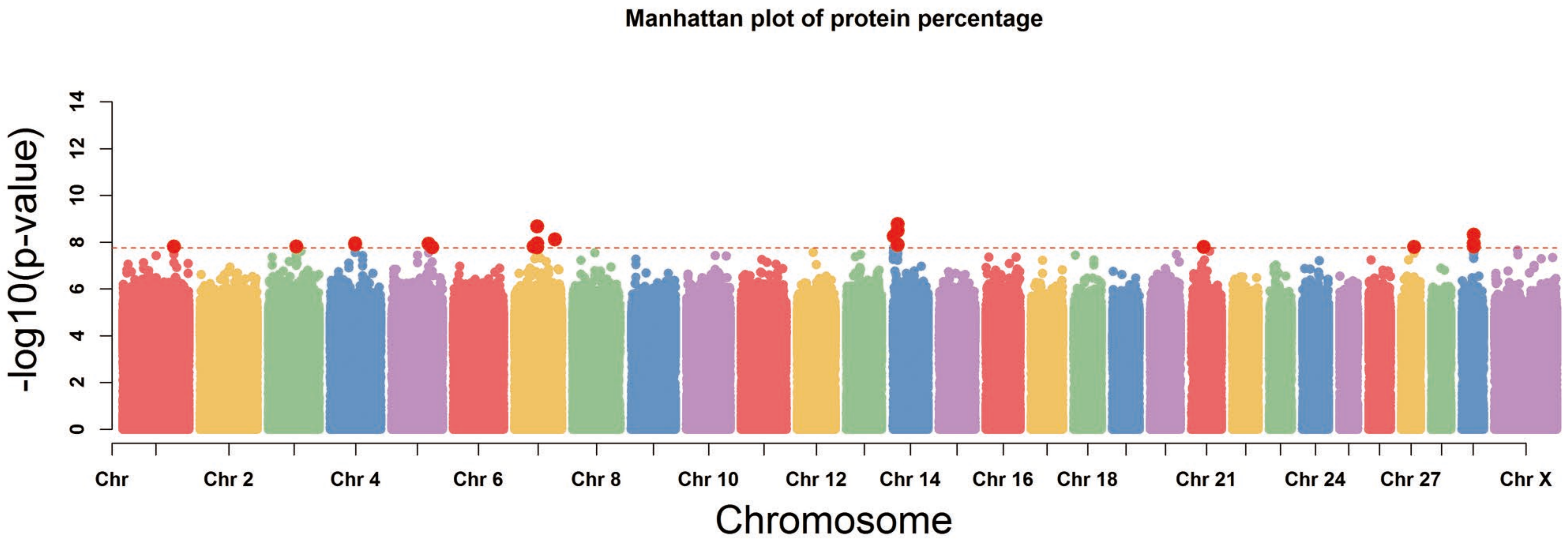

Fig. S17. Manhattan plot for the –log10 (P-value) of single SNP associated with protein percentage

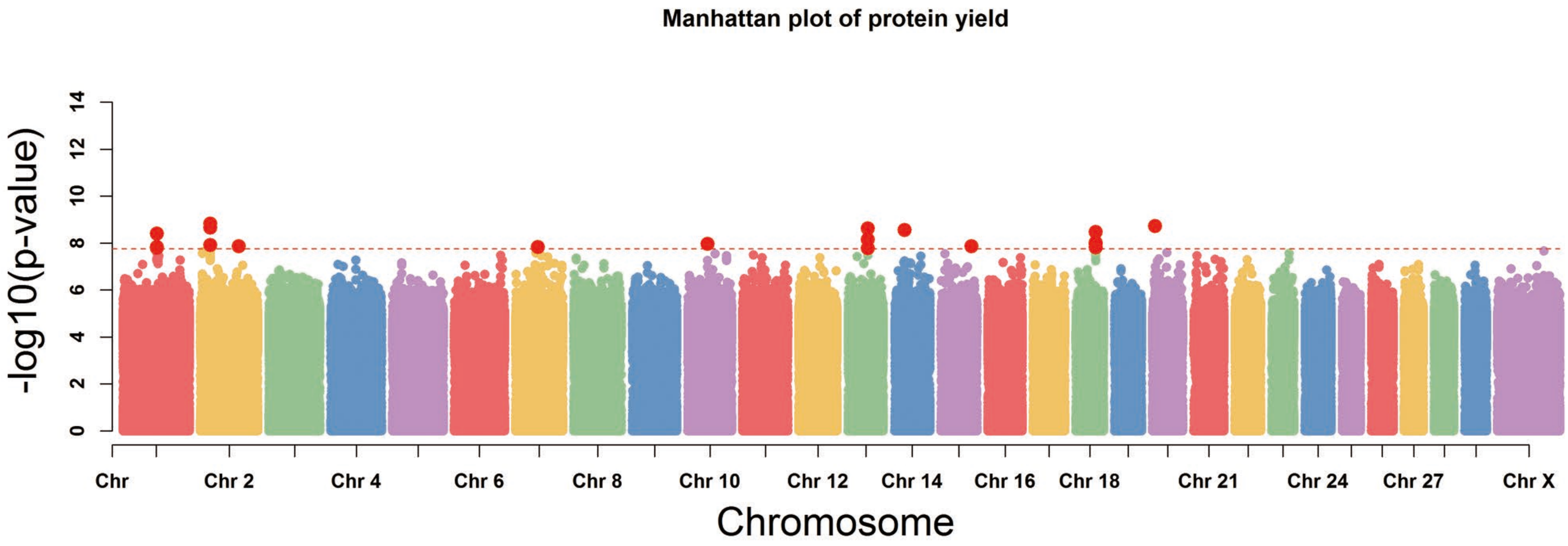

Fig. S18. Manhattan plot for the –log10 (P-value) of single SNP associated with protein yield

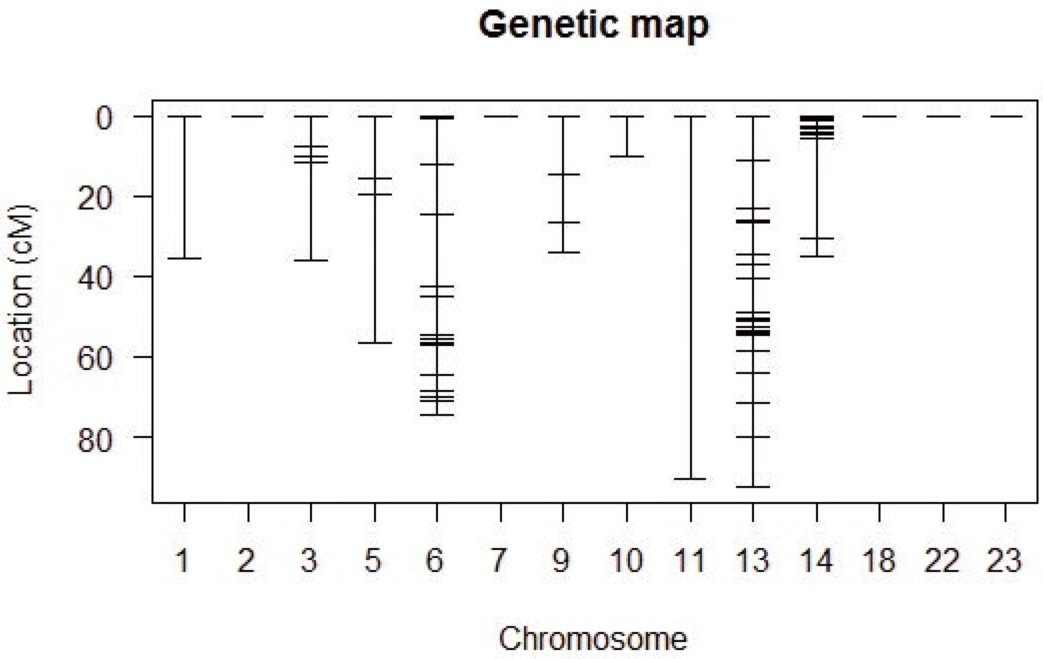

Fig. S19. Genetic map for 118 QTL related to milk protein composition
